# Effectiveness of home treatment in children and adolescents with psychiatric disorders—systematic review and meta-analysis

**DOI:** 10.1186/s12916-024-03448-2

**Published:** 2024-06-13

**Authors:** Daniel Graf, Christine Sigrist, Isabel Boege, Marialuisa Cavelti, Julian Koenig, Michael Kaess

**Affiliations:** 1 University Hospital of Child and Adolescent Psychiatry and Psychotherapy, University of Bern, Bern, Switzerland; 2 Department of Child and Adolescent Psychiatry, Psychosomatics and Psychotherapy, Faculty of Medicine and University Hospital Cologne, University of Cologne, Cologne, Germany; 3 Department of Child and Adolescent Psychiatry, ZfP Suedwuerttemberg, Ravensburg, Germany; 4 Department of Child and Adolescent Psychiatry, Center for Psychosocial Medicine, University Hospital Heidelberg, Heidelberg, Germany

**Keywords:** Home treatment, Treatment setting, Child and adolescent psychiatry, Treatment research, Meta-analysis

## Abstract

**Background:**

Home treatment in child and adolescent psychiatry offers an alternative to conventional inpatient treatment by involving the patient’s family, school, and peers more directly in therapy. Although several reviews have summarised existing home treatment programmes, evidence of their effectiveness remains limited and data synthesis is lacking.

**Methods:**

We conducted a meta-analysis on the effectiveness of home treatment compared with inpatient treatment in child and adolescent psychiatry, based on a systematic search of four databases (PubMed, CINAHL, PsychINFO, Embase). Primary outcomes were psychosocial functioning and psychopathology. Additional outcomes included treatment satisfaction, duration, costs, and readmission rates. Group differences were expressed as standardised mean differences (SMD) in change scores. We used three-level random-effects meta-analysis and meta-regression and conducted both superiority and non-inferiority testing.

**Results:**

We included 30 studies from 13 non-overlapping samples, providing data from 1795 individuals (mean age: 11.95 ± 2.33 years; 42.5% female). We found no significant differences between home and inpatient treatment for postline psychosocial functioning (SMD = 0.05 [− 0.18; 0.30], *p* = 0.68, *I*^2^ = 98.0%) and psychopathology (SMD = 0.10 [− 0.17; 0.37], *p* = 0.44, *I*^2^ = 98.3%). Similar results were observed from follow-up data and non-inferiority testing. Meta-regression showed better outcomes for patient groups with higher levels of psychopathology at baseline and favoured home treatment over inpatient treatment when only randomised controlled trials were considered.

**Conclusions:**

This meta-analysis found no evidence that home treatment is less effective than conventional inpatient treatment, highlighting its potential as an effective alternative in child and adolescent psychiatry. The generalisability of these findings is reduced by limitations in the existing literature, and further research is needed to better understand which patients benefit most from home treatment.

**Trial registration:**

Registered at PROSPERO (CRD42020177558), July 5, 2020.

**Supplementary Information:**

The online version contains supplementary material available at 10.1186/s12916-024-03448-2.

## Background

Most mental disorders have their onset in childhood or adolescence [1, 2], with global point prevalence estimates at nearly 14% in this young population [3]. Recent research suggests that the global COVID-19 pandemic in early 2020 has contributed to an increase in the prevalence of affective, eating, and anxiety disorders, as well as in emergencies involving self-harm [4–7]. Simultaneously, the pandemic has increased the media presence of mental health in young people, reducing the stigma associated with mental disorders [8] and promoting more positive attitudes toward seeking professional help [9]. Both of these factors contribute to growing waiting lists for admission to inpatient treatment (IT) [10–12], exacerbating a long-standing problem in child and adolescent psychiatry [13, 14].

Home treatment (HT) is not new to the field of child and adolescent psychiatry but is becoming increasingly important to address these challenges promising a possible alternative to IT that can be more rapidly implemented and scaled up. Different to IT, the young patients remain in their home environment and are visited on a frequent and regular basis by a multi-professional treatment team, including child and adolescent psychiatrists and psychotherapists, social workers, and nursing staff. The close involvement of the patient’s family, school, and the broader social environment (e.g. peers) in therapy allows problems to be observed and addressed where they arise, holding the potential to increase sustainability of treatment effects and reduced readmission rates [15, 16]. Furthermore, HT has been suggested to be more cost-effective than IT [17], supported by two studies in the general child and adolescent psychiatry using acceptability curves based on QALYs [18] and the incremental cost-effectiveness ratios (ICER) based on changes in the psychosocial functioning [19]. Consequently, HT could allow treatment to be offered to a greater number of patients at the same cost.

These considerations of HT, its rationale, and implementation in general psychiatry date back to the 1960s [20]. In child and adolescent psychiatry, HT programmes were implemented as early as the 1970s and 1980s in the USA [21] and Europe [22]. Further clinical trials followed over the last four decades and several reviews were published, providing an overview of the consistently growing body of literature [23–28]. These reviews highlight the potential of HT as a promising alternative to IT; however, their conclusions are limited by the sparse underlying evidence and the small study samples. In addition, to the best of our knowledge, no meta-analysis of trials examining the effectiveness of HT in child and adolescent psychiatry has been conducted, as done previously for adult psychiatry [29, 30].

To close this gap, we updated the most recent literature searches on this topic in 2020 [23, 27] and conducted a meta-analysis to investigate the effectiveness of HT as an alternative to IT for children and adolescents with mental disorders. In addition, we sought to explore patient subgroups that are more likely to benefit from HT, taking into account various demographic and contextual variables.

## Methods

This systematic review and meta-analysis followed the PRISMA guidelines [31] (checklist in Additional file 1, pp. 2–4). The study protocol was registered at PROSPERO (registration CRD42020177558).

### Search strategy and selection criteria

We systematically searched PubMed, CINAHL, PsychINFO, and Embase for relevant articles in April 2020, with two updates in December 2022 and December 2023 (search strategy detailed in Additional file 1, Table S2). Additionally, we performed manual backward and forward snowballing of the reference lists of included articles and contacted the authors of all included studies to inquire about other potential HT trials or experts in the field. We did not search grey literature or trial registries. One rater (DG) screened titles and abstracts for inclusion/exclusion criteria, followed by full-text screening, using the Rayyan web application for systematic reviews [32]. To test robustness of the screening process, a random 10% sample of identified records was screened by a second rater (SE). The decisions for inclusion or exclusion were in complete agreement. Full texts were obtained online, through interlibrary loan [33], and from antiquarian bookshops [22, 34]. The inclusion criteria were as follows: empirical clinical trials published in English- or German-language journals or books; intervention: HT equivalent to IT and presence of a control group receiving IT or equivalent care; population: patients with psychiatric diagnoses; mean age ≤ 21 years. Non-randomised controlled trials (nRCTs) were included due to the previously reported paucity of randomised controlled trials (RCTs) in this research area [24] and concerns about the generalisability of RCTs to real-world contexts [30].

### Experimental and control treatment

Although recent literature provides more clarity and consensus regarding the nature and scope of intensive community care services [35], “home treatment” was often used in the past (and still is used) as an umbrella term for treatments delivered in a home-based setting, including supported discharge service (SDS) [36], Home-Based Crisis Intervention (HBCI) [37], Multisystemic Therapy (MST) [38], and others [30]. In the present study, we defined HT as an intensive psychiatric treatment delivered in a home-based setting that was intended to entirely replace or shorten an inpatient stay (“equivalent” to IT) [30, 39]. Treatment programmes with different names that met the above criteria were considered HT (e.g. MST as an alternative to hospitalisation) [38]. The key element of all HT programmes was that they offered treatment outside of the clinic, which would have been the alternative treatment. Therapy sessions were primarily conducted at the patient’s home but additional options such as school visits or assistance with daily activities like using public transport or grocery shopping were often available. Presence of day services such as day clinic or group therapy carried out in the clinic was no criterion for excluding a HT programme, provided the majority of the treatment took place in the home environment. We defined IT as treatment delivered in a hospital ward or similar institutional setting, including residential care [40].

### Choice of primary and secondary outcome

The primary outcomes were psychosocial functioning and psychopathology. These outcomes are considered relevant for daily life functioning, also from the perspective of youth with lived experience [41], and sensitive to changes over the course of treatment. Secondary outcomes included treatment cost, duration, and satisfaction. Where appropriate, we combined similar outcome measures from different instruments and studies (e.g. different instruments assessing “psychosocial functioning”). Details on the grouping of instruments are provided in the Additional file 1 (pp. 5–7). Outcome measures were categorised according to their source of information (clinician-rated, self-rated, parent-rated).

### Data extraction and processing

Two reviewers (DG and SO) independently extracted information about the treatments (description, duration, intensity), study population (sample size, dropouts, age and sex distribution, primary psychiatric diagnoses), study design (randomisation, timing of endpoints), and outcome measures for each group and time of assessment (i.e. *n*, *M*, *SD/var*). If relevant data was not reported in the studies, we contacted the authors to obtain the information (response rate: 50%) or derived it by calculation of other data reported in the article (Additional file 1, p. 8).

### Risk of bias assessment

We assessed the methodological risk of bias using the “Cochrane Collaboration Risk of Bias 2.0” (ROB2) [42] for RCTs and the “Risk Of Bias In Non-randomised Studies—of Interventions” (ROBINS-I) [43] for nRCTs. RCTs were categorised as having low, medium, or high risk of bias based on the following criteria: randomisation process, deviations from planned interventions, missing outcome data, outcome measurement, and selection of reported outcomes. nRCTs were classified as having low, moderate, serious, or critical risk of bias based on the following criteria: confounding, selection of study participants, classification of interventions, deviations from planned interventions, missing data, measurement of outcomes, and selection of reported results.

### Calculation of effect size measures

We calculated the standardised mean difference (SMD) for each outcome as the effect size measure, comparing HT to IT based on the difference between baseline and (a) postline values or (b) follow-up values, if available. For RCT studies, we employed formulas proposed by Becker [44] and Carlson and Schmidt [45] as described in Morris [46] to estimate SMD (*d*_ppc_). Due to the common scenario of unknown correlation between pre- and post-treatment measures in meta-analysis, we assumed *ρ* = 0.50. For nRCT studies, meta-analytic procedures were adjusted to account for the precision of effect sizes. For each study, the difference between the sample means at post-treatment or follow-up was divided by the pooled standard deviation at baseline and corrected for small-sample bias [47]. The exact formulas were used in this calculation of Hedges’ *g* and corresponding standard errors [48]. Readmission rates reported as percentages were translated to a 2 × 2 frequency table, based on which respective log odds ratios were calculated [49, 50]. For studies reporting mean readmissions, SMDs were calculated and converted into log odds ratios (e.g. [51–54]), which were back-transformed into regular odds ratios (OR) for better interpretability after data synthesis. An OR above 1 indicated a higher rate of readmission after IT compared to HT, whereas an OR below 1 indicated the opposite.

### Data synthesis

In most cases, effect sizes were nested within clusters of individual study samples based on rater perspective and time of assessment. That is, separate meta-analyses were conducted for post-treatment and follow-up effects. Clustering was specified for rater perspective for primary outcomes and treatment satisfaction, and for time of measurement for treatment costs. Three-level random-effects meta-analytical models [55], which allow effect sizes to vary between participants (level 1), outcomes (level 2), and studies (level 3) [56], were used to synthesise the cluster effects. We used inverse variance weighting and a restricted maximum likelihood estimator (REML) to estimate level 2 and level 3 *τ*^2^ values. Heterogeneity was assessed using a generalised/weighted least squares extension of Cochran’s test [57]. For the synthesis of the treatment duration data, a conventional (two-level) meta-analytical model was used given the lack of clustering in these data. Inverse variance weighting and REML were used to estimate level 2 *τ*^2^. Confidence intervals for individual studies and tests of individual coefficients and confidence intervals were calculated based on a *t*-distribution (with degrees of freedom), such that the omnibus test used an *F*-distribution [58]. Forest plots were used to visualise meta-analytical summary models for outcome, and funnel plots were used to visually explore asymmetry. We conducted data analysis using the R-packages “meta” and “metafor” [57, 59].

### Moderator analyses

Meta-regression analyses were conducted to separately examine the potentially moderating effects of various factors on the effectiveness of HT compared with IT, including mean age (in years), sex (% female), mean duration of treatment (in days), study design (RCT vs. nRCT), type of HT (adjunctive to IT vs. substitute for IT), and presence of day services (provided during HT vs. not provided). Baseline scores of the primary outcomes were considered both as pooled mean scores to test whether generally higher or lower levels influenced post-treatment outcomes and as the difference in means (Δ = *M*_HT_ − *M*_IT_) to account for differences between groups at the onset of treatment, which can be expected particularly in nRCTs. Multivariate meta-analytical models tested continuous and categorical moderators using an omnibus test (*QM* test) [57]. If a particular moderator was missing, the corresponding study was excluded from the meta-regression analyses. It is important to note that the meta-regression analyses are exploratory in nature and that the results should be interpreted with caution due to the potential for overfitting when the number of studies per covariate examined is small [60]. For the same reason, meta-regression analysis was conducted only for the primary outcomes of psychosocial functioning and psychopathology.

### Objective non-inferiority assessment of primary outcomes

Considering that HT as a “novel” treatment is unlikely to be *superior* to IT from a real-world clinical perspective, we additionally conducted *non-inferiority testing* in the meta-analyses of primary outcomes as proposed by Trone et al. [61]. Non-inferiority testing evaluates whether a novel treatment is not worse than the comparator by the degree of “acceptable inferiority”, defined by the non-inferiority margin (∆) based on the reported effect of the active comparator. First, the effect size and corresponding 95% confidence interval (CI) of the active comparator versus an untreated control group (SMD_Inptr_) were determined. Given the lack of evidence in the literature (i.e. no existing meta-analysis examined the efficacy of IT vs. untreated control), we performed an additional systematic search (detailed in Additional file 1, pp. 9–10) to obtain the effect size (95% CI) of IT for each primary outcome. We defined 50% and 95% as the percentage (alpha) of the effect of IT to test whether the effect was maintained with HT. ∆ was calculated using SMD_Inptr_ and the upper bound of the 95% CI of SMD_Inptr_, respectively (with the latter being the more conservative approach to calculating an objective non-inferiority margin). After calculating ∆, we compared the 95% CI of the summary effect size of HT versus IT for primary outcomes obtained from meta-analysis of the respective RCTs, with the non-inferiority margin (∆). To demonstrate non-inferiority, the 95% CI of the HT vs. IT comparison should fall entirely on the left (negative) side of ∆.

## Results

**Fig. 1:**
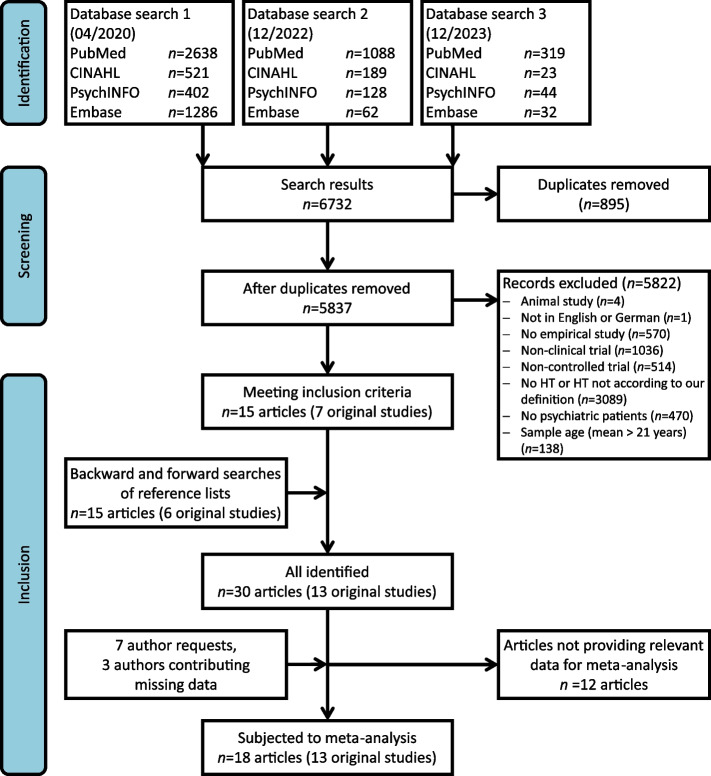
PRISMA flowchart of the systematic search

Our search strategy yielded a total of 4072 unique records from the original search (04/2020) and 1735 additional from two literature update (12/2022 and 12/2023). The PRISMA flowchart in Fig. 1 summarises the selection procedure, which resulted in the inclusion of 28 articles and two books. These 30 publications reported relevant data from 13 non-overlapping samples comprising 1795 individuals (average baseline age: 11.95 ± 2.33 years; 42.5% female).

All included trials are summarised in Table 1. They were conducted in Europe (*k* = 8, 61.5%), the USA (*k* = 3, 23.1%), and Canada (*k* = 2, 15.4%). The majority of the trials used HT to entirely replace IT (*k* = 9, 69.2%) and assigned patients randomly to the treatment groups (*k* = 8, 61.5%). Risk of bias assessments showed moderate-to-high risk for most RCTs and all nRCTs (Additional file 1, Figures S2 and S3).

**Table 1:** Characteristics of the included publications. Studies referring to the same sample are clustered within sections; bolded studies were included in the meta-analysis

	**No. of patients**^**a**^	**Age range (*M* ± *SD*)**	**Female No. (%)**	**Study design**	**Diagnoses**	**HT condition (intensity), duration (*M* ± *SD*)**	**Control condition, duration (*M* ± *SD*)**	**Outcome measures**	**Endpoints**	**Risk of bias**
Boege et al. (2014) [62] Germany	92	5–17 (13.7 ± 2.8)	49 (53.3%)	RCT	General psychiatric disorders	Short IT with early discharge and subsequent HT (Ø40.4 h with family), day services in the clinic could be used during HT, duration IT: 47.7 ± 28.8 days, duration HT: 109 days	IT, duration: 69.4 ± 30.7 days	K-SADS, CIS, HoNOSCA, CGAS, SDQ	Discharge	Some concerns
**Boege et al. (2015)** [19]								CGAS, costs in €	Discharge Follow-up (8 months)	Some concerns
**Boege et al. (2015)** [63]								K-SADS, CIS, HoNOSCA, CGAS, SDQ	Discharge	Some concerns
**Kirchmann et al. (2014))** [64]								BesT	Discharge Follow-up (8 months)	Some concerns
**Boege et al. (2021))** [65]								CGAS, HoNOSCA	Discharge Follow-up 1 (8 months), follow-up 2 (48 months)	Some concerns
**Henggeler et al. (1999)** [38] USA	113	10–17 (12.9 ± 2.1)	39 (34.5%)	RCT	Psychiatric emergencies, general psychiatric disorders	Multisystemic Therapy (Ø97.1 h with family), no treatment elements in the clinic during HT, duration: 123.0 ± 29.0 days	Hospitalisation, duration: 5.8 ± 3.5 days	CBCL, PEI, DISC, GSI-BSI, FFS, FACES-III, LFSS	Discharge inpatient/discharge HT	Some concerns
**Schoenwald et al. (2000)** [66]								Service Utilisation Survey, Restrictiveness of Living Environments Scale, hospital records	Discharge inpatient/discharge HT	Some concerns
Henggeler et al. (2003) [67]								CBCL, DISC, GSI-BSI, FFS, FACES-III, Service Utilisation Survey	Discharge inpatient/discharge HT Follow-up 1 (6 months) Follow-up 2 (12 months)	Some concerns
Sheidow et al. (2004) [68]								Costs in $, CBCL, GSI	Discharge inpatient/discharge HT Follow-up 2 (12 months)	Some concerns
Huey et al. (2004) [69]								FFS, CBCL, GSI-BSI, YRBS	Discharge inpatient/discharge HT Follow-up 2 (12 months)	Some concerns
**Mattejat et al. (2001)** [70] Germany	68	6–17 (11.1 ± 3.3)	24 (35.3%)	RCT	10 specified diagnoses (~ 15% of all inpatients treated in that clinic)	HT (intensity not described), approx. ¼ of contacts took place in the clinic during HT, duration: 120.9 days	IT, duration: 90.5 days	MSS, rating of psychosocial competency	Discharge Follow-up (44 months)	High
Remschmidt et al. (1988) [33]								MVL, CBCL, categorisation in improvement/no change/deterioration	Discharge	High
Remschmidt (1988) [34]								MVL, CBCL, 7-point Likert scale for psychosocial competence, treatment satisfaction	Discharge	High
**Ougrin et al. (2018)** [18] UK	106	12–17 (16.3 ± 1.6)	69 (65.1%)	RCT	General psychiatric disorders	Short IT with early discharge and subsequent supported discharge service including HT (SDS, intensity flexible, up to a maximum of daily contacts), day services in hospital could be used during HT, duration: 116.3 ± 70.1 days	IT, duration: 50.0 days	SDQ, CGAS, SHQ, ChASE; costs in £, Child and Adolescent Service Use Schedule	6 months post randomisation	Low
**Ougrin et al. (2021)** [71]								CIS, HoNOSCA, SHQ, CGI-I	6 months post randomisation	Low
**Reimer (1983)** [22] Germany	62	8–12 (n/a)	13 (21.0%)	RCT	Not specified	HT (Ø38 contacts with family), no treatment elements in the clinic during HT, duration: 90.0 days	IT, duration: 90.0 days	AFS, PFK, conflicting behaviour between child and parent questionnaire (21 items), performance/social/anxiety questionnaire (39 items)	Discharge Follow-up (6 months)	Some concerns—high
**Schmidt et al.** (2006) [16] Germany	105	6–17 (11.0 ± 3.0)	36 (34.3%)	nRCT	General psychiatric disorders (no extreme rare diagnoses)	HT (Ø20 contacts with family, 3 contacts with relevant others), no treatment elements in the clinic during HT, duration: 105.0 ± 21.0 days	IT, duration: 105.0 ± 42.0 days	SGKJ, MEI, MAS, 7-point Likert scale for psychosocial functioning, 7-point Likert scale for changes in symptoms	Discharge Follow-up (13.7 months)	Moderate
Schmidt et al. (1998) [17]								Not reported	Discharge, preliminary data	Moderate
**Winsberg et al.** (1980) [21] USA	49	5–13 (9.4 ± 1.4)	8 (16.3%)	RCT	General psychiatric disorders (i.e. emotional and behaviour disorders)	Short IT with early discharge and subsequent HT (intensity not described), no treatment elements in the clinic during HT, duration IT: 7–21 days, duration HT: 177 days	IT, duration: 138.0 days	BRS, DCB, DESB, MAT, SESAT, PSS, FFC	Discharge	High
**Evans et al. (2003)** [37] USA	238	5–18 (12.3 ± 3.6)	112 (47.1%)	RCT	General psychiatric disorders	Home-Based Crisis Intervention and Enhanced Home-Based Crisis Intervention (intensity not described), no treatment elements in the clinic during HT, duration: 4–6 weeks	Crisis Case Management, duration: 4–6 weeks	CAFAS, FACES-II, CBCL, Piers-Harris Children’s Self Concept Scale	Discharge Follow-up (6 months)	Some concerns
**Preyde, Frensch, et al. (2011)** [40] Canada	169	6–18 (11.6 ± 2.8)	42^b^ (24.9%)	nRCT	General psychiatric disorders	HT (~ 10 h per week), no treatment elements in the clinic during HT, duration: 157.5 ± 108.0 days	Residential treatment centres, 24-h facilities that are not licensed as a hospital but do offer supervision and mental health treatment programmes for children, duration: 234.0 ± 174.0 days	CAFAS, BCFPI	Discharge Follow-up 1 (12–18 months) Follow-up 2 (36–40 months)	Serious
Preyde, Cameron, et al. (2011) [72]								CAFAS, BCFPI, KINDL, FAD	Discharge Follow-up (12–18 months)	Serious
Cameron et al. (2011) [73]								CAFAS, BCFPI	Discharge Follow-up (12–18 months)	Serious
Preyde et al. (2010) [74]								CAFAS, BCFPI	Discharge Follow-up (12–18 months)	Serious
Frensch et al. (2009) [75]								CAFAS, BCFPI, KINDL	Discharge Follow-up (12–18 months)	Serious
Preyde et al. (2009) [76]								CAFAS, BCFPI	Discharge Follow-up (12–18 months), preliminary data	Serious
**Graf et al. (2021)** [77] Switzerland	132	6–17 (13.7 ± 2.9)	71 (53.8%)	nRCT	General psychiatric disorders	HT (daily contacts except of weekends), no treatment elements in the clinic during HT, duration: 83.7 ± 28.0 days	IT, duration:100.8 ± 62.7 days	HoNOSCA, GAF, treatment satisfaction questionnaire	Discharge	Serious
**Herpertz-Dahlmann et al. (2020)** [78], **Herpertz-Dahlmann et al. (2014)** [79], for control group Germany	106	14–17 (15.2 ± 1.4)	106 (100%)	Independent samples	Anorexia nervosa	Short IT with early discharge and subsequent HT (Ø4.4 contacts per week during the first month), no treatment elements in the clinic during HT, duration IT: 53.2 ± 6.7 days, duration HT: 108.5 days	IT, duration: 102.2 days	EDI-2, MRAOS, BMI^c^	Discharge Follow-up (12 months)	Critical
**Wilmshurst (2002)** [80] Canada	65	6–14 (10.7 ± 2.1)	11^b^ (16.9%)	RCT	General psychiatric disorders	HT (Ø48.3 ± 15.0 h), no treatment elements in the clinic during HT, duration: 90.0 days	Residential treatment centres, 24-h facilities that are not licensed as a hospital but do offer supervision and mental health treatment programmes for children, duration: 90.0 days	SCIS, SSRS	Discharge Follow-up (12 months)	High
**Erkolahti et al. (2004)** [81] Finland	490	3–13 (9.1 ± 1.66)	181 (37%)	nRCT	General psychiatric disorders	HT (not specified, ranged from once a week to once a month), day services in hospital could be used during HT, duration: not reported	IT, duration: not reported	CGAS	Discharge	Serious

*Abbreviations: HT* Home treatment, *IT *Inpatient treatment; *nRCT* non-randomised controlled trial, *RCT* Randomised controlled trial, *AFS* Angstfragebogen für Schüler, *BCFPI* Brief child and family phone interview, *BesT* Behandlungseinschätzung stationär-psychiatrischer therapie, *BMI* Body mass index, *BRS* Conners behaviour rating scale; *CAFAS* Child and adolescent functional assessment scales, *ChASE* Child and adolescent service experience, *CBCL* Child behaviour checklist, *YRBS* Youth risk behaviour survey, *CGAS* Children’s global assessment scale, *CGAS* Children’s global assessment scale, *CGI-I* Clinical global impression—Improvement scale, *CIS* Columbia Impairment scale, *DCB* Devereux child behaviour rating scale, *DESB* Devereux elementary school behaviour rating scale, *DISC* Diagnostic interview schedule for children, *EDI-2* Eating disorder inventory-2, *FACES-III* Family adaptability and cohesion evaluation scales, *FAD* Family assessment device, *FFC* Family functioning checklist, *FFS* Family friends and self scale, *GSI-BSI* Global severity index of the brief symptom inventory, *HoNOSCA* Health of the nations outcome scale for children and adolescents, *KINDL* Quality of life questionnaire, *K-SADS* Kiddie-schedule for affective disorders and schizophrenia, *LFSS* Lubrecht’s family satisfaction survey, *MAS* Multiaxial classification scheme for psychiatric diseases in children and adolescents, *MAT* Metropolitan achievement test, *MEI* Mannheim parent interview, *MRAOS *Morgan and russell average outcome score, *MSS *Marburg symptom scale, *MVL* Marburger verhaltensliste, *PEI* Personal experiences inventory, *PFK* Persönlichkeitsfragebogen für kinder, *PSS* Psychiatric status schedule, *SCIS* Standardised client information system, *SDQ* Strength and difficulties questionnaire, *SDQ* Strengths and difficulties questionnaire, *SESAT* Stanford early school achievement test, *SGKJ* Global assessment scale for children and adolescents, *SHQ* Self-harm questionnaire, *SSRS* Social skills rating system

^a^Number of patients providing relevant data, dropouts excluded

^b^number of the study sample not reported and therefore estimated based on the study population; include dropouts throughout treatment

^c^outcomes that were assessed in both intervention and control group only

### Psychosocial functioning

**Fig. 2:**
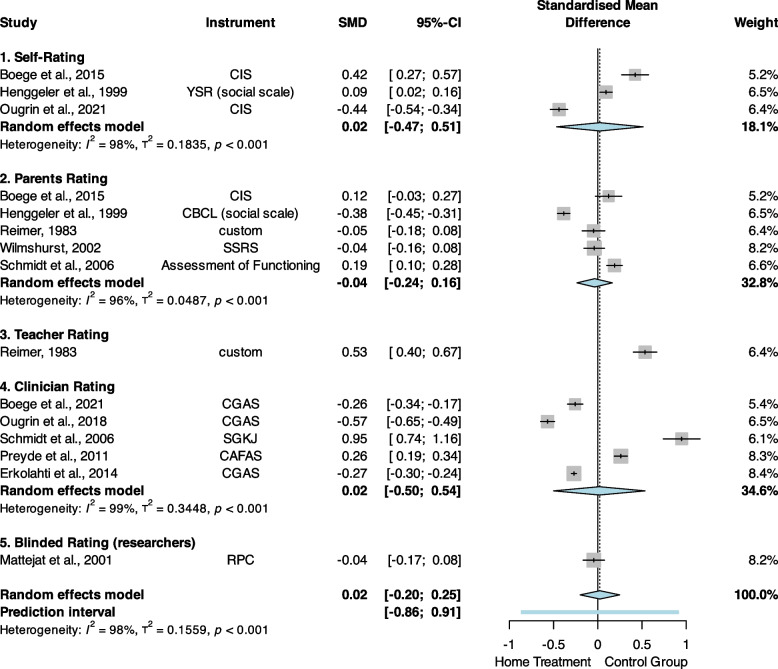
Differences in pre- to post-treatment effects in psychosocial functioning scores. SMD, standardised mean difference; CAFAS, Child and Adolescent Functioning Assessment Scale; CBCL, Child Behaviour Checklist; CGAS, Children’s Global Assessment Scale; CIS, Columbia Impairment Scale; RPC, rating of psychosocial competency; SGKJ, global assessment scale for children and adolescents (“Skala zur Gesamtbeurteilung von Kindern und Jugendlichen”); SSRS, Social Skills Rating System; YSR, Youth Self-Report

For the primary outcome of psychosocial functioning, we excluded one study [21] from the analysis, because the outcomes for the two treatment groups were assessed by two independent rater groups that differed substantially in their ratings. The forest plot in Fig. 2 shows the individual and summary effect size estimates. The final pooled effect size of postline assessments (*n* = 9 studies,* k* = 15 estimates, *N* = 1722) was SMD = 0.02 [95% CI, − 0.20 to 0.25], *p* = 0.83. Overall heterogeneity was substantial, with *I*^2^ = 98.1% ([95% CI, 97.6% to 98.5%], *Q*_14_ = 751.48,* p* < 0.001). Visual inspection of the corresponding funnel plots (Additional file 1, Figure S4) suggested the presence of small study bias and one clear outlier [16]. The meta-regression analyses did not identify any significant moderators (Additional file 1, Table S7).

For follow-up assessments (*n* = 5 studies, *k* = 7 estimates, *N* = 516), the pooled effect size was SMD =  − 0.15 [95% CI, − 0.39 to 0.09], *p* = 0.23 (Additional file 1, Figure S5). Overall heterogeneity was substantial, with *I*^2^ = 95.0% ([95% CI, 91.9% to 96.9%], *Q*_6_ = 119.75, *p* < 0.001). Sensitivity analyses by type of design did not alter these results (Additional file 1, Figures S6–S8).

### Psychopathology

**Fig. 3:**
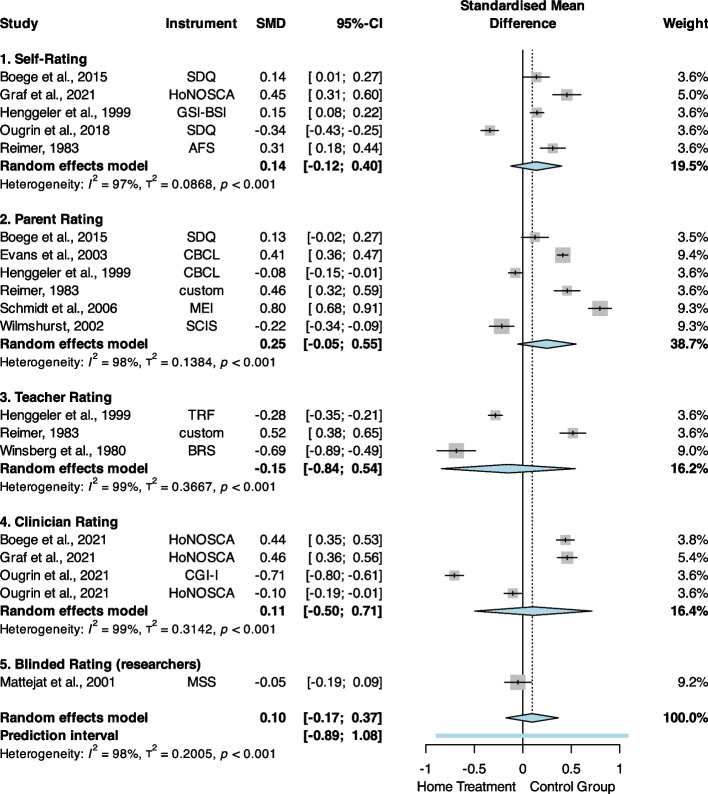
Differences in pre- to post-treatment effects in psychopathology. SMD, standardised mean difference; AFS, anxiety questionnaire for pupils (“Angstfragebogen für Schüler”); BRS, Conners Behaviour Rating Scale; CBCL, Child Behaviour Checklist; CGI-I, Clinical Global Impression—Improvement scale; GSI-BSI, Global Severity Index of the Brief Symptom Inventory; HoNOSCA, Health of the Nations Outcome Scale for children and adolescent; MEI, Mannheim Parents Interview (“Mannheimer Eltern Interview”); MSS, Marburg Symptom Scale; SCIS, Standardised Client Information System; SDQ, Strength and Difficulties Questionnaire; TRF, Teacher Report Form

Regarding the primary outcome of psychopathology, we excluded one study [78] from the data synthesis, because the data from this study was compared to that of another study conducted years earlier with a different sample [79]. Prior to the exclusion of this study, overall quality/risk of bias was identified as a significant moderator of the summary effect size, which was no longer the case after this study was excluded, suggesting that it introduced bias into the respective meta-analysis. The forest plot in Fig. 3 illustrates the individual and summary effect size estimates. The resulting pooled effect size of postline assessments (*n* = 10 studies,* k* = 19 estimates, *N* = 1629) was SMD = 0.01 [95% CI, − 0.17 to 0.37], *p* = 0.48. Overall heterogeneity was substantial, with *I*^2^ = 98.3% ([95% CI, 98.0% to 98.6%], *Q*_19_ = 1083.61, *p* < 0.001). Visual inspection of the corresponding funnel plots (Additional file 1, Figure S4) suggested no clear study bias, but the presence of one outlier [21].

**Fig. 4:**
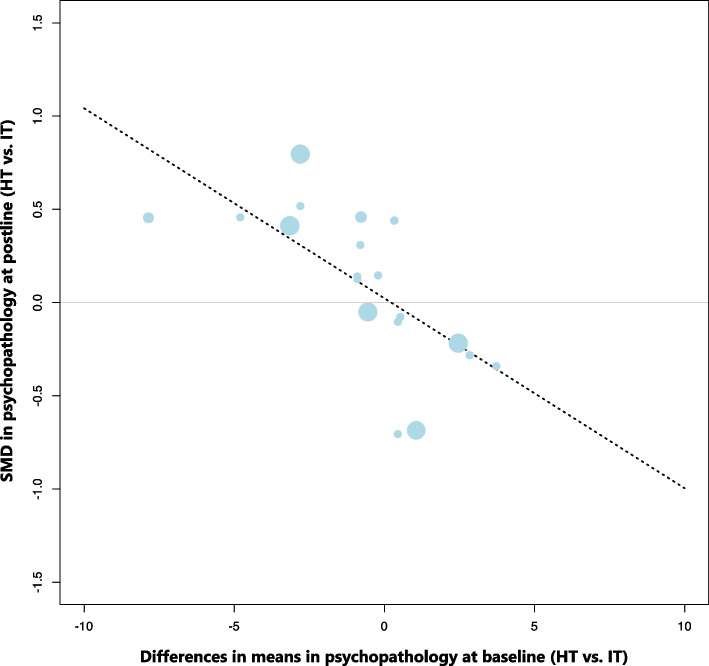
Meta-regression scatterplot showing the association between baseline differences in means in psychopathology and standardised mean differences (SMD) at postline. Positive delta scores indicate higher baseline psychopathology in the HT group compared to the IT group; negative SMD favour HT at postline

Meta-regression analyses showed that differences in mean scores at baseline (*k* = 19, *β* =  − 0.10, [95% CI, − 0.16 to − 0.05], SE = 0.03, *p* < 0.001) and the study design (*k* = 19, *β* =  − 0.64, [95% CI, − 1.21 to − 0.07], SE = 0.29, *p* = 0.03) significantly moderated the individual effect size estimates. On average, effect sizes increased for patient groups with higher levels of psychopathology at baseline (relative to the other group, see Fig. 4) and tended to favour HT over IT when only RCTs were considered (Additional file 1, Table S7).

For follow-up assessments, the pooled effect size (*n* = 7 studies, *k* = 9 estimates, *N* = 749) was SMD = 0.05 [95% CI, − 0.18 to 0.27], *p* = 0.69 (Additional file 1, Figure S9). Overall heterogeneity was substantial, with *I*^2^ = 95.8% ([95% CI, 93.8% to 97.2%], *Q*_8_ = 192.09, *p* < 0.001).

Notably, one study [37] compared HT with another alternative for IT (“Crisis Case Management”), which met the formal inclusion criteria but differed substantially from the control condition we intended for comparison as no inpatient or residential care was involved. A sensitivity analysis excluding this study showed negligible differences from the overall meta-analysis (Additional file 1, Figures S10 and S11), as did a sensitivity analysis considering only RCTs (Additional file 1, Figures S12 and S13). When considering only nRCTs, the resulting pooled effect size of postline assessments (*n* = 2 studies,* k* = 3 estimates, *N* = 304) was SMD = 0.62 [95% CI, 0.29 to 0.96], *p* = 0.002 (*I*^2^ = 90.7%, [95% CI, 75.7% to 96.5%], *Q*_2_ = 21.55, *p* < 0.001; see Additional file 1, Figure S14); the result for follow-up outcomes did not change (Additional file 1, Figure S15).

### Secondary outcomes

Regarding the treatment satisfaction, the pooled effect size (*n* = 4 studies,* k* = 7 estimates, *N* = 529) was SMD = 0.08 [95% CI, − 0.70 to 0.86], *p* = 0.84. Overall heterogeneity was substantial, with *I*^2^ = 99.0% ([95% CI, 98.7% to 99.3%], *Q*_6_ = 606.61, *p* < 0.001).

For treatment duration, the pooled effect size (*n* = 5 studies, *N* = 491) was SMD =  − 1.73 [95% CI, − 3.92 to 0.46], *p* = 0*.*12. Overall heterogeneity was substantial, with *I*^2^ = 99.7% ([95% CI, 99.6% to 99.8%], *Q*_4_ = 1356.38, *p* < 0.001).

Regarding treatment costs, the pooled effect size (*n* = 2 studies, *k* = 3 estimates, *N* = 290, one study [68] was not considered due to inconsistent reporting) was SMD =  − 1.55 [95% CI, − 4.56 to 1.46], *p* = 0.313. Overall heterogeneity was substantial, with *I*^2^ = 99.9% ([95% CI, 99.8% to 99.9%], *Q*_4_ = 1559.47, *p* < 0.001).

For readmission rates, the pooled effect size (*n* = 3 studies, *k* = 3 estimates) was OR = 1.27 (95% CI, 0.74 to 2.18, *p* = 0.39) with no significant heterogeneity observed (*I*^2^ < 0.01%, *Q*_2_ = 1.60, *p* = 0.45). Forest plots for all secondary outcomes are provided in Additional file 1, Figures S16–S19.

### Non-inferiority testing

The systematic search for the efficacy of conventional IT for youth with mental disorders yielded two studies [82, 83]. The resulting SMD was 0.64 [95% CI, 0.60 to 0.68] for psychosocial functioning (*n* = 1 study, *k* = 1 estimate, *N* = 150) and 0.27 [95% CI, 0.08 to 0.46] for psychopathology (*n* = 1 study, *k* = 2 estimates, *N* = 132). The calculated objective non-inferiority margins for each primary outcome are shown in Table 2, along with the SMD between HT and IT for each primary outcome based on RCT studies.

**Table 2:** Results of the non-inferiority testing

Outcome	Endpoint				Objective non-inferiority margin			
	**SMD**_**Inptr**_	**[95% CI]**	**SMD**_**HTvsInpt**_	**[95% CI]**	**Δ**_**50%**_	**Δ**^**MAX**^_**50%**_	**Δ**_**95%**_	**Δ**^**MAX**^_**95%**_
Psychosocial functioning	0.64	0.60; 0.68	− 0.06	− 0.29; 0.16	1.25	1.21	1.02	1.02
Psychopathology	0.27	0.08; 0.46	− 0.03	− 0.29; 0.24	1.92	1.48	1.07	1.04

*Abbreviations*: *SMD*_*Inptr*_ Standardised mean difference between IT and untreated control per primary outcome, *SMD*_*HTvsInpt*_ Standardised mean difference between HT and IT per primary outcome based on RCT studies, *Δ*_*50%*_* and Δ*^*MAX*^_*50% *_Non-inferiority margins (50% of the effect of conventional psychiatric IT, according to the value of SMD_Inptr_, and of its 95% CI upper bound, respectively), *Δ*_*95%*_* and Δ*^*MAX*^_*95%*_ non-inferiority margins corresponding to 95% of the effect of conventional psychiatric IT, according to the value of SMD_Inptr_, and the value of its 95% CI upper bound, respectively

Evidence of non-inferiority of HT was obtained for both primary outcomes of psychosocial functioning and psychopathology. First, conventional IT resulted in a significant improvement in the primary outcomes compared with no treatment (waitlist controls). Second, regardless of the non-inferiority margin used (i.e. 50% or 95%; based on SMD_Inptr_ or the respective upper bound of the 95% CI), HT appeared to be non-inferior to conventional IT. Figure S20 in Additional file 1 illustrates the results of the non-inferiority assessment and Figures S21 and S22 show the forest plots based on the non-inferiority analysis.

## Discussion

The aim of this meta-analysis was to synthesise the existing data on the effectiveness of HT as an alternative to IT for youth with mental disorders. Based on a comprehensive synthesis of 30 articles (18 providing relevant data) derived from 13 non-overlapping samples with a total of 1795 individuals, we examined differences in treatment outcomes including potential moderators.

Our analyses for both superiority and non-inferiority testing showed no significant postline differences between patients who received HT and those who received IT with respect to the primary outcomes psychosocial functioning and psychopathology. This finding is consistent with conclusions drawn in several previous reviews of the existing data, suggesting that HT is generally not less effective than conventional IT [24, 27, 28].

The mean difference between groups at baseline was identified as a significant moderator of post-treatment psychopathology: on average, patient groups with higher levels of psychopathology at baseline (relative to the other group) showed greater improvements in the postline outcome (expressed as a higher SMD). Both IT and HT appear to be particularly effective for patients with severe psychopathological burden, for whom both services are designed. Alternatively, this effect may also reflect a regression to the mean as patients presenting with higher levels of psychopathology at baseline presumably had greater potential for improvement during treatment compared to those with lower baseline levels. Study design moderated post-treatment psychopathology, with effect sizes favouring HT over IT when only RCTs were considered and sensitivity analysis with only nRCTs showed significantly better psychopathology outcomes at postline for IT. This emphasises the importance of using rigorous methodological approaches in evaluation studies. In RCTs, treatments are usually delivered according to a strict protocol, ensuring high treatment fidelity. HT, as implemented in RCTs, might be more standardised and thus more effective compared to more variable programmes in less controlled study designs. Besides, patients who participated in RCTs may have hoped to be assigned to the HT group. Their disappointment when randomised to the control group may have affected their expectations of treatment, which has been associated with negative treatment outcome [84]. However, given the modest number of studies included in the meta-regression analyses and their exploratory nature, these findings should be considered indicative rather than conclusive and should be interpreted with caution, highlighting areas where further research is needed to support them. Despite the expectation that HT would be less expensive because of the reduced reliance on clinic infrastructure and staff, we found no significant difference in treatment costs between HT and IT. Possible explanations include the hospitalisation of some patients during the course of the HT and the fact that certain HT programmes compensated for lower intensity with longer treatment duration. However, the total duration of treatment was not significantly different between the two modalities. Furthermore, and contrary to expectations, readmission rates after discharge did not differ significantly between the two treatment settings. These findings do not support the expectation that HT is a cheaper alternative and leads to fewer readmissions due to a better transfer of treatment gains after discharge in HT.

However, the conclusions drawn from these findings are limited by the small sample sizes, with only two studies included in the meta-analysis of treatment costs [18, 19] and three studies in the meta-analysis of readmission rates [65, 71, 78]. A direct comparison of the overall cost-effectiveness of the two treatments was not possible due to insufficient data.

This meta-analysis adheres to several aspects of good practice, including the pre-registration of a review protocol, considerable effort to obtain all available data (including contacting interlibrary loan, antiquarian booksellers, and authors of all studies), double‐rated data extraction by two independent reviewers, and the use of objective non-inferiority testing for primary outcomes.

However, our findings should be viewed in the context of several limitations, concerning both our methodology and the existing body of literature. We found considerable statistical heterogeneity in all results, reflecting our broad interpretation of the term “home treatment”. In nine studies, HT completely replaced hospitalisation [16, 21, 22, 37, 38, 40, 70, 77, 80], while in the other four, it only reduced the length of hospital stay [18, 62, 78, 81]. Moreover, while most studies strictly separated the home and clinical environments, some provided additional day services during HT. These included distinct treatment elements such as structured daily routines, group therapy and opportunities for bonding with other patients, which have also been reported as important in the treatment of children and adolescents with psychiatric disorders [85, 86]. The intensity of HT also varied widely, ranging from a maximum of 12 h per week [80] to a minimum of one visit per month [81], and while most programmes addressed general psychopathology, two targeted specific diagnoses [33, 78]. Inconsistencies between studies in the selected outcomes and the instruments used to measure them may have introduced additional heterogeneity into the results, as may the combination of RCTs and nRCTs, which could also have affected the overall null effect. Although we conducted sensitivity analyses by types of design, these results should be interpreted with caution due to the small number of studies per subgroup. Besides, the generally small number of individual studies for the meta-regression analyses should also be noted. Meta-regression models can be overfitted when the number of studies per covariate examined is small, which may lead to spurious associations between covariates and treatment effect due to data idiosyncrasies [60]. Thus, these analyses need to be considered exploratory and interpreted with caution. For psychosocial functioning, only nine studies were included, which is below the minimum of 10 as suggested in the Cochrane Handbook [87]. However, there is also evidence that the required number of observations per covariate in ordinary least squares linear regression might be considerably lower than 10 [60]. We chose to explore potential moderators for effect size in this outcome, as such analyses can provide important information about directions for future research.

In terms of the search strategy, restricting our search to PubMed, CINAHL, PsychINFO, and Embase may have led to the omission of some relevant studies. The search results were screened by a single rater only with a second-rater screening for a random 10% sample to test the robustness of the process. The decision for inclusion or exclusion was in complete agreement; however, this approach leaves an increased risk of overlooking relevant studies in the remaining search results.

Regarding the available evidence, the small number of eligible studies, many of which used small samples, limited the statistical power, especially for secondary outcomes not reported in all studies. This made it impossible to further specify the treatment characteristics of the included HT to reduce heterogeneity. The moderate to high risk of bias in twelve out of thirteen studies indicates an overall low study quality. Additionally, the diversity of the studies, spanning four decades and six countries (all located in Europe and North America) with different legal and financial frameworks, as well as varying IT quality, limits the generalisability of our findings to other healthcare systems. Most studies did not explore potential mechanisms underlying the effectiveness of HT, such as the involvement of the whole (family) system, and left open the question of which family situations and diagnostic patterns are more likely to benefit from HT.

To address these limitations and replicate the current findings, further research on HT in child and adolescent psychiatry, as well as meta-analysis of its results as more studies are published, is urgently needed. Future studies should consider some important aspects: to ensure standardised treatment designs in future studies, it is advisable to refer to current guidelines, such as the agreed minimum requirements proposed by Keiller et al. [35]. Moreover, we suggest focusing on a set of key constructs including psychosocial functioning, psychiatric symptoms, quality of life, family functioning, and patient satisfaction to streamline the diversity in outcome measures. For consistent and comparative measurement, researchers may consult current reviews of widely used, reliable and validated instruments (e.g. Kwan and Rickwood [88] or the International Consortium for Health Outcomes Measurements [89]). Cost-effectiveness of new programmes should not only consider direct treatment costs, but also subsequent psychiatric care, such as inpatient readmissions, emergency department visits, medication, and outpatient treatments post-discharge. Quantifying the contacts with patients, families, peers, and schools during the HT could help understanding the potential mechanisms underlying its effectiveness and to explore the influence of systemic and individual factors in presenting disorders. Our study also highlights the importance of stringent methodological designs in treatment evaluation. This involves the use of randomised control groups and assessments at multiple time points (pre-, post-treatment, and follow-up), executed by trained and blinded researchers. If randomisation is difficult to realise due to health economic factors like imbalances in treatment group capacities, adaptive randomisation plans might be considered.

However, adhering to these methodological standards often requires additional resources, such as research staff or strategies for handling patient allocation disparities. Therefore, we call upon policymakers to not only endorse future HT projects in clinical practice but also support their scientific evaluation.

## Conclusions

In this meta-analysis, we found no evidence that HT is generally less effective than conventional IT. Both treatments appear to be particularly effective in patients with a high psychopathological burden, highlighting the potential of HT as an effective alternative to IT in child and adolescent psychiatry. However, the generalisability of these findings is restricted by various limitations in the existing literature, and several unanswered questions remain. Further research is needed to identify patients who are more likely to benefit from HT based on their family situation and diagnosis patterns.

### Supplementary Information

Supplementary Material 1: Table S1. PRISMA 2020 Checklist. Table S2. Detailed search strategy. Table S3-S5. Grouping of different instruments. Table S6. Listing of all data derived by calculation. Figure S1. Additional systematic search on the efficacy of Inpatient Treatment. Figure S2 & S3. Summary of the risk of bias of RCTs and nRCTs. Figure S4. Funnel plots of individual observed effect sizes. Table S7. Meta-regression results. Figure S5. Follow-up effects in psychosocial functioning. Figure S6-S8. Sensitivity analyses for psychosocial functioning, including only RCTs or nRCTs. Figure S9. Follow-up effects in psychopathology. Figure S10 & S11. Sensitivity analyses for psychopathology excluding the study of Evans et al. (2003). Figure S12-S15. Sensitivity analyses for psychopathology, including only RCTs or nRCTs. Figure S16-S19. Meta-analyses of secondary outcomes. Figure S20-S22. Meta-analyses based on non-inferiority assessments.

## Data Availability

The underlying dataset and analysis code used in this article [90] are available without restrictions on the Open Science Framework (osf.io) and can be found here: 10.17605/OSF.IO/TFD2Q.

## Abbreviations

CI: Confidence interval
HT: Home treatment
HBCI: Home-based crisis intervention
IT: Inpatient treatment
MST: Multisystemic therapy
nRCT: Non-randomised controlled trial
OR: Odds ratio
PRISMA: Preferred reporting items for systematic reviews and meta-analyses
RCT: Randomised controlled trial
REML: Restricted maximum likelihood estimator
ROB2: Cochrane collaboration risk of bias 2.0
ROBINS-I: Risk of bias in non-randomised studies – of interventions
SE: Standard error
SMD: Standardized mean difference

## Declarations

### Declarations

Ethics approval and consent to participate.

Not applicable.
