# Supplementary material for: Effectiveness of home treatment in children and adolescents with psychiatric disorders—systematic review and meta-analysis
Source: BMC Med. 2024 Jun 13;22:241. doi: 10.1186/s12916-024-03448-2 (PMC11170798; doi:10.1186/s12916-024-03448-2)
Supplement: Supplementary file 1 — Supplementary Material 1: Table S1. PRISMA 2020 Checklist. Table S2. Detailed search strategy. Table S3-S5. Grouping of different instruments. Table S6. Listing of all data derived by calculation. Figure S1. Additional systematic search on the efficacy of Inpatient Treatment. Figure S2 & S3. Summary of the risk of bias of RCTs and nRCTs. Figure S4. Funnel plots of individual observed effect sizes. Table S7. Meta-regression results. Figure S5. Follow-up effects in psychosocial functioning. Figure S6-S8. Sensitivity analyses for psychosocial functioning, including only RCTs or nRCTs. Figure S9. Follow-up effects in psychopathology. Figure S10 & S11. Sensitivity analyses for psychopathology excluding the study of Evans et al. (2003). Figure S12-S15. Sensitivity analyses for psychopathology, including only RCTs or nRCTs. Figure S16-S19. Meta-analyses of secondary outcomes. Figure S20-S22. Meta-analyses based on non-inferiority assessments. [file 12916_2024_3448_MOESM1_ESM.docx]

**Supplementary Information**

Supplement to Graf, Sigrist, Boege, Cavelti, Koenig, Kaess. Home Treatment for Children and Adolescents with Psychiatric Disorders – A Systematic Review and Meta-Analysis.

Table of Supplementary Information

[Table S1. PRISMA 2020 Checklist 2](#_Toc166336968)

[Supplementary Methods 5](#_Toc166336969)

[Table S2. Detailed search strategy 5](#_Toc166336970)

[Grouping of different instruments 5](#_Toc166336971)

[Table S3. Instruments considered for secondary outcomes 5](#_Toc166336972)

[Table S4. Instruments considered for psychosocial functioning 6](#_Toc166336973)

[Table S5. Instruments considered for psychopathology 7](#_Toc166336974)

[Documentation of score calculations 8](#_Toc166336975)

[Table S6. Listing of all data derived by calculation 8](#_Toc166336976)

[Additional systematic search on the efficacy of Inpatient Treatment 9](#_Toc166336977)

[Figure S1. Prisma flowchart of the additional systematic search 10](#_Toc166336978)

[Supplementary Results 11](#_Toc166336979)

[Figure S2. Summary of the risk of bias of RCTs 11](#_Toc166336980)

[Figure S3. Summary of the risk of bias of nRCTs 11](#_Toc166336981)

[Figure S4. Funnel plots of individual observed effect sizes 12](#_Toc166336982)

[Table S7. Meta-regression results (conducted for primary outcomes) 13](#_Toc166336983)

[Figure S5. Difference in pre- to follow-up effects in psychosocial functioning 14](#_Toc166336984)

[Figure S6. Sensitivity analysis: pre- to post-treatment effects in psychosocial functioning, including only Randomised Controlled Trials (RCTs) 14](#_Toc166336985)

[Figure S7. Sensitivity analysis: pre- to follow-up effects in psychosocial functioning, including only Randomised Controlled Trials (RCTs) 15](#_Toc166336986)

[Figure S8. Sensitivity analysis: pre- to post-treatment effects in psychosocial functioning, including only non-Randomised Controlled Trials (nRCTs) 15](#_Toc166336987)

[Figure S9. Difference in pre- to follow-up effects in psychopathology 16](#_Toc166336988)

[Figure S10. Sensitivity analysis: pre- to post-treatment effects in psychopathology, excluding the study of Evans et al. (2003) [37] 17](#_Toc166336989)

[Figure S11. Sensitivity analysis: pre- to follow-up effects in psychopathology, excluding the study of Evans et al. (2003) [37] 18](#_Toc166336990)

[Figure S12. Sensitivity analysis: pre- to post-treatment effects in psychopathology, including only Randomised Controlled Trials (RCTs) 19](#_Toc166336991)

[Figure S13. Sensitivity analysis: pre- to follow-up effects in psychopathology, including only Randomised Controlled Trials (RCTs) 20](#_Toc166336992)

[Figure S14. Sensitivity analysis: pre- to post-treatment effects in psychopathology, including only non-Randomised Controlled Trials (nRCTs) 20](#_Toc166336993)

[Figure S15. Sensitivity analysis: pre- to follow-up effects in psychopathology, including only non-Randomised Controlled Trials (nRCTs) 21](#_Toc166336994)

[Figure S16. Difference in treatment satisfaction (post-treatment) 21](#_Toc166336995)

[Figure S17. Difference in treatment duration (in days) 21](#_Toc166336996)

[Figure S18. Difference in treatment costs 22](#_Toc166336997)

[Figure S19. Difference in readmission rates post-discharge 22](#_Toc166336998)

[Figure S20. Non-inferiority asssessment of Home Treatment (HT) vs. Inpatient Treatment (IT) for Psychosocial Functioning and Psychopathology 23](#_Toc166336999)

[Figure S21. Non-inferiority assessment: differences in pre- to post-treatment effects in psychosocial functioning 24](#_Toc166337000)

[Figure S22. Non-inferiority assessment: differences in pre- to post-treatment effects in in psychopathology 25](#_Toc166337001)

# Table S1. PRISMA 2020 Checklist

| **Section and Topic** | **Item #** | **Checklist item** | **Location where item is reported** |
| --- | --- | --- | --- |
| **TITLE** | | |  |
| Title | 1 | Identify the report as a systematic review. | p.1 |
| **ABSTRACT** | | |  |
| Abstract | 2 | See the PRISMA 2020 for Abstracts checklist. | pp.2-3 |
| **INTRODUCTION** | | |  |
| Rationale | 3 | Describe the rationale for the review in the context of existing knowledge. | p.4 |
| Objectives | 4 | Provide an explicit statement of the objective(s) or question(s) the review addresses. | p.5 |
| **METHODS** | | |  |
| Eligibility criteria | 5 | Specify the inclusion and exclusion criteria for the review and how studies were grouped for the syntheses. | p.6 |
| Information sources | 6 | Specify all databases, registers, websites, organisations, reference lists and other sources searched or consulted to identify studies. Specify the date when each source was last searched or consulted. | p.5 |
| Search strategy | 7 | Present the full search strategies for all databases, registers and websites, including any filters and limits used. | p.5, Table S2 |
| Selection process | 8 | Specify the methods used to decide whether a study met the inclusion criteria of the review, including how many reviewers screened each record and each report retrieved, whether they worked independently, and if applicable, details of automation tools used in the process. | pp.5-6 |
| Data collection process | 9 | Specify the methods used to collect data from reports, including how many reviewers collected data from each report, whether they worked independently, any processes for obtaining or confirming data from study investigators, and if applicable, details of automation tools used in the process. | pp.6-7 |
| Data items | 10a | List and define all outcomes for which data were sought. Specify whether all results that were compatible with each outcome domain in each study were sought (e.g. for all measures, time points, analyses), and if not, the methods used to decide which results to collect. | p.6, Suppl. Methods p.5;  Table S3, Table S4**;** Table S5 |
|  | 10b | List and define all other variables for which data were sought (e.g. participant and intervention characteristics, funding sources). Describe any assumptions made about any missing or unclear information. | p.7 |
| Study risk of bias assessment | 11 | Specify the methods used to assess risk of bias in the included studies, including details of the tool(s) used, how many reviewers assessed each study and whether they worked independently, and if applicable, details of automation tools used in the process. | pp.7-8 |
| Effect measures | 12 | Specify for each outcome the effect measure(s) (e.g. risk ratio, mean difference) used in the synthesis or presentation of results. | p.8 |
| Synthesis methods | 13a | Describe the processes used to decide which studies were eligible for each synthesis (e.g. tabulating the study intervention characteristics and comparing against the planned groups for each synthesis (item #5)). | p.8 |
|  | 13b | Describe any methods required to prepare the data for presentation or synthesis, such as handling of missing summary statistics, or data conversions. | p.8, Suppl. Methods p.8, Table S6 |
|  | 13c | Describe any methods used to tabulate or visually display results of individual studies and syntheses. | p.8 |
|  | 13d | Describe any methods used to synthesize results and provide a rationale for the choice(s). If meta-analysis was performed, describe the model(s), method(s) to identify the presence and extent of statistical heterogeneity, and software package(s) used. | pp.8-11 |
|  | 13e | Describe any methods used to explore possible causes of heterogeneity among study results (e.g. subgroup analysis, meta-regression). | pp.9-10 |
|  | 13f | Describe any sensitivity analyses conducted to assess robustness of the synthesized results. | pp.12-14 |
| Reporting bias assessment | 14 | Describe any methods used to assess risk of bias due to missing results in a synthesis (arising from reporting biases). | p.9 |
| Certainty assessment | 15 | Describe any methods used to assess certainty (or confidence) in the body of evidence for an outcome. | - |
| **RESULTS** | | |  |
| Study selection | 16a | Describe the results of the search and selection process, from the number of records identified in the search to the number of studies included in the review, ideally using a flow diagram. | p.11, Figure 1 |
|  | 16b | Cite studies that might appear to meet the inclusion criteria, but which were excluded, and explain why they were excluded. | Figure 1 |
| Study characteristics | 17 | Cite each included study and present its characteristics. | Table 1 |
| Risk of bias in studies | 18 | Present assessments of risk of bias for each included study. | Table 1, Figure S2**,** Figure S3 |
| Results of individual studies | 19 | For all outcomes, present, for each study: (a) summary statistics for each group (where appropriate) and (b) an effect estimate and its precision (e.g. confidence/credible interval), ideally using structured tables or plots. | Figure 2, Figure 3, Figure S5**,** Figure S9**,** Figure S16**,** Figure S17**,** Figure S18**,** Figure S19**,** Figure S20**,** Figure S21**,** Figure S22 |
| Results of syntheses | 20a | For each synthesis, briefly summarise the characteristics and risk of bias among contributing studies. | p.11 |
|  | 20b | Present results of all statistical syntheses conducted. If meta-analysis was done, present for each the summary estimate and its precision (e.g. confidence/credible interval) and measures of statistical heterogeneity. If comparing groups, describe the direction of the effect. | pp.12-15 |
|  | 20c | Present results of all investigations of possible causes of heterogeneity among study results. | pp.12-14, Figure 4, Table S7 |
|  | 20d | Present results of all sensitivity analyses conducted to assess the robustness of the synthesized results. | Figure S6, Figure S7, Figure S8, Figure S10**,** Figure S11**,** Figure S12**,** Figure S13**,** Figure S14**,** Figure S15 |
| Reporting biases | 21 | Present assessments of risk of bias due to missing results (arising from reporting biases) for each synthesis assessed. | Figure S4 |
| Certainty of evidence | 22 | Present assessments of certainty (or confidence) in the body of evidence for each outcome assessed. | - |
| **DISCUSSION** | | |  |
| Discussion | 23a | Provide a general interpretation of the results in the context of other evidence. | pp.15-16 |
|  | 23b | Discuss any limitations of the evidence included in the review. | pp.18-19 |
|  | 23c | Discuss any limitations of the review processes used. | pp.17-18 |
|  | 23d | Discuss implications of the results for practice, policy, and future research. | pp.19-20 |
| **OTHER INFORMATION** | | |  |
| Registration and protocol | 24a | Provide registration information for the review, including register name and registration number, or state that the review was not registered. | p.3 |
|  | 24b | Indicate where the review protocol can be accessed, or state that a protocol was not prepared. | p.3 |
|  | 24c | Describe and explain any amendments to information provided at registration or in the protocol. | - |
| Support | 25 | Describe sources of financial or non-financial support for the review, and the role of the funders or sponsors in the review. | p.21 |
| Competing interests | 26 | Declare any competing interests of review authors. | p.21 |
| Availability of data, code and other materials | 27 | Report which of the following are publicly available and where they can be found: template data collection forms; data extracted from included studies; data used for all analyses; analytic code; any other materials used in the review. | pp.22-23 |

# Supplementary Methods

#### **Table S2.** Detailed search strategy

|  | **Search string** |
| --- | --- |
| a) | (child* OR adolescen* OR teen*) |
| AND |  |
| b) | (mental OR psych*) |
| AND |  |
| c) | (home* OR mobil* OR community OR outreach) |
| Filters | PubMed: Clinical Trial; Abstract; Humans  CINAHL: Abstract Available; Human; Publication Type: Clinical Trial  PsychINFO: human and abstracts and "0300 clinical trial"  Embase: abstracts and human and controlled clinical trial |

## Grouping of different instruments

Many of the included studies used different instruments to assess the same construct (e.g., psychosocial functioning). Therefore, we paid particular attention to the description and classification of the instruments that were combined in the meta-analysis. In the case of an unclear outcome classification, consensus was reached through discussion between DG, JK, and MK. One outcome was excluded from the analysis because it could not be clearly classified as psychosocial functioning or psychopathological outcome (Piers-Harris Children’s Self Concept Scale in Evans et al. (2003)) [37]. When outcome measures were coded inversely (e.g., psychosocial functioning vs. psychosocial malfunctioning), we recoded them to ensure comparability. Table S4 provides an overview of all instruments considered for the meta-regression of psychosocial functioning. Table S5 of all instruments for psychopathological outcomes and Table S3 of all instruments for the secondary outcomes.

#### **Table S3.** Instruments considered for secondary outcomes

| **Study** | **Instrument** | **Completed by** | **Description** |
| --- | --- | --- | --- |
| **Treatment satisfaction** | | | |
| Kirchmann et al. (2014) [64] | BesT | Patient, parent | *Behandlungseinschätzung stationär-psychiatrischer Therapie*; consists of 36 items completed by the patients and 29 items completed by the parents to assess overall satisfaction with the treatment and its effectiveness. |
| Henggeler et al. (1999) [38] | LFSS | Patient, parent | *Lubrecht’s Family Satisfaction Survey*; no further information trackable. |
| Ougrin et al. (2018) [18] | ChASE | Patient | *Child and Adolescent Service Experience;* consists of 22 items, assessing core expectations about therapy, the content of appointments, process of therapy, and expected outcomes. |
| Winsberg et al. (1980) [21]^a^ | PFI | Parents | *Parent’s Final Impression*; custom open-ended questions, dichotomized: satisfied/not satisfied. |
| Graf et al. (2021) [77] | Custom | Patient, parent | *Treatment satisfaction questionnaire*; consists of 24 items, assessing satisfaction with initiation and completion phase, transparency, treatment, and team. |
| **Cost** | | | |
| Boege et al. (2015) [19] | | | Direct costs in € |
| Schoenwald et al. (2000) [66]^b^ | | | Direct costs in $ |
| Ougrin et al. (2018) [18] | | | Direct costs in £ |

^a^not included in the meta-analysis due to dichotomous data; ^b^not included in the meta-analysis due to inconsistent reporting.

#### **Table S4.** Instruments considered for psychosocial functioning

| **Study** | **Instrument** | **Completed by** | **Description** |
| --- | --- | --- | --- |
| Boege et al. (2014) [62] | CIS | Parents, patient | *Columbia Impairment Scale*; assesses psychosocial impairment with 13 items on 4 domains: interpersonal relationships, psychopathological dimensions, school/work behaviour, leisure behaviour |
| Boege et al. (2021) [65],  Ougrin et al. (2018) [18], Erkolahti et al. (2004) [81] | CGAS | Clinician | *Children’s Global Assessment Scale*; global measure to assess general functioning as a single rating on a scale from 1 to 100 |
| Henggeler et al. (1999) [38] | CBCL | Parents | *Child* Behaviour *Checklist*; consists of 118 items to assess childhood externalizing and internalizing disorders. *Social Competence* subscale was used as indicator of psychosocial functioning |
| Henggeler et al. (1999) [38] | YSR | Patient | *Youth Self-Report*; parallel form to the CBCL completed by the youth, consists of 118 items to assess externalizing and internalizing disorders. *Social Competence* subscale was used as indicator of psychosocial functioning |
| Mattejat et al. (2001) [70] | RPC | Blinded rater | *Rating of psychosocial competency*; assesses three domains with one item each: social relationships, school/work adjustment, and leisure time activities on 7-point Likert items |
| Preyde et al. (2011) [40] | CAFAS | Clinician | *Child and Adolescent Functioning Assessment Scale*; assesses psychosocial functioning along eight domains: role performance at school or work, home, community (e.g., delinquent acts), behaviour toward others, moods/ emotions (e.g., anxiety, depression), self-harm behaviour, substance use, and problems in thinking |
| Reimer (1983) [22] | Custom Scale | Parents, teacher | 37 items, assessing three domains: performance behaviour, social behaviour/antisocial behaviour, (social) anxiety/neuroticism. S*ocial* behaviour*/antisocial* behaviour subscale was used as indicator of psychosocial functioning |
| Schmidt et al. (2006) [16] | Custom Scale | Parents | Assesses five domains: family, performances, peer relationships, interests, and autonomy on 7-point Likert items |
| Schmidt et al. (2006) [16] | SGKJ | Clinician | *Global assessment scale for children and adolescents* (“*Skala zur Gesamtbeurteilung von Kindern und Jugendlichen*”); adaptation of the CGAS, global measure to assess general functioning as a single rating on a scale from 1 to 10 |
| Wilmshurst (2002) [80] | SSRS | Parent | *Social Skills Rating System*; assesses social competence in domains of cooperation, assertion, self-control, and responsibility |

#### **Table S5.** Instruments considered for psychopathology

| **Study** | **Instrument** | **Completed by** | **Description** |
| --- | --- | --- | --- |
| Reimer (1983) [22] | AFS | Patient | *Anxiety questionnaire for pupils* (“*Angstfragebogen für Schüler*”); assesses manifest anxiety, test anxiety, school dislike, social desirability |
| Winsberg et al. (1980) [21] | BRS | Teacher | *Conners* Behaviour *Rating Scale*; consists of 39 items to assess disordered behaviour in five domains: aggressivity, inattentiveness, anxiety, hyperactivity, and sociability |
| Evans et al. (2003) [37],  Henggeler et al. (2003) [17] | CBCL | Parents | *Child* Behaviour *Checklist*; consists of 118 items to assess childhood externalizing and internalizing disorders |
| Ougrin et al. (2021) [71] | CGI-I | Clinician | *Clinical Global Impression – Improvement scale*; assesses general clinical improvement |
| Reimer (1983) [22] | Custom Scale | Parents, teacher | 37 items, assessing three domains: performance behaviour, social behaviour/antisocial behaviour, (social) anxiety/neuroticism. *(Social) anxiety/neuroticism* subscale was used as indicator of internalizing psychopathological symptoms |
| Henggeler et al. (1999) [38] | GSI | Patient | *Global Severity Index*; computed from the 53 items of the BSI (*Brief Symptom Inventory*) as a measure of emotional distress |
| Boege et al. (2021) [65],  Ougrin et al. (2021) [71],  Graf et al. (2021) [77] | HoNOSCA | Patient, clinician | *Health of the Nations Outcome Scale for children and adolescent*; consist of 13 items to assess a range of behavioural, symptomatic, social, and impairment domains and provide a global outcome for psychopathology |
| Schmidt et al. (2006) [16] | MEI | Parents | *Mannheim Parents Interview* (“Mannheimer Eltern Interview”); consists of 40 items to assess psychopathological symptoms |
| Mattejat et al. (2001) [70] | MSS | Blinded rater | *Marburg Symptom Scale*; consists of 22 items to assess psychopathological symptoms |
| Wilmshurst (2002) [80] | SCIS | Parents | *Standardized Client Information System*; consists of 60 items originating from the CBCL to assess emotional and behavioural disorders |
| Boege et al. (2015) [19],  Ougrin et al. (2018) [18] | SDQ | Patient, parent | *Strength and Difficulties Questionnaire*; consists of 25 items, covering five domains: emotional symptoms, conduct problems, hyperactivity/inattention, peer relationship problems, prosocial behaviour |
| Henggeler et al. (1999) [38] | TRF | Teacher | *Teacher Report Form*; parallel form to the CBCL completed by the teacher, consists of 118 items to assess childhood externalizing and internalizing disorders |

## Documentation of score calculations

When data were not presented in a form suitable for meta-analysis, we contacted the authors of the original publications and asked them to provide the necessary statistics or the raw data, and then calculated the relevant outcomes ourselves. Moreover, we retrieved fragmentary data by calculating the available data when possible (e.g., by pooling different subscales of a functioning instrument into an overall functioning score). Table S6 provides an overview of all scores derived by calculation rather than direct extraction. For full calculations, please contact the authors. Data for all extracted and calculated scores, as well as the code for the meta-analysis, will be made available in the Open Science Framework (osf.io).

#### **Table S6.** Listing of all data derived by calculation

| **Study** | **Description** |
| --- | --- |
| Boege et al. (2021) [65] | Raw data was provided by the authors, *M* & *SD* were computed for CIS, SDQ, CGAS, & HoNOSCA |
| Henggeler et al. (1999) [38] | Pooling *M* & *SD* of the CBCL internalizing & externalizing subscales |
| Mattejat et al. (2001) [70] | Pooling *M* & *SD* of the Mannheim and the Marburg sample of the MMS and “Adaptation at School or Work” |
| Schmidt et al. (2006) [16] | Pooling *M* & *SD* of five subscales (family, achievement, peers, interests, autonomy) into an overall psychosocial functioning score |
| Winsberg et al. (1980) [21] | Pooling *M* & *SD* of the three subscales (Aggressivity/Inattentiveness/Hyperactivity) of psychopathological symptoms to one score,  pooling *M* & *SD* of age data from HT & IT group |
| Herpertz-Dahlmann et al. (2020) [78],  Herpertz-Dahlmann et al. (2014) [79] | Pooling *M* & *SD* of age data from the two different samples |
| Henggeler et al. (1999) [38] | Pooling *M* & *SD* of internalizing and externalizing disorder subscales of the SCIS into an overall psychopathological outcome |
| Wilmshurst (2002) [80] | Pooling *M* & *SD* of age data from HT & IT group |

Abbreviations: *M*=Mean; *SD*=Standard Deviation; CBCL=Child Behaviour Checklist; CGAS=Children’s Global Assessment Scale; CIS=Columbia Impairment Scale; HoNOSCA=Health of the Nations Outcome Scale for children and adolescents; MSS=Marburg Symptom Scale; MMS=Marburg Symptom Scale; SDQ=Strengths and Difficulties Questionnaire; SCIS=Standardized Client Information System.

## Additional systematic search on the efficacy of Inpatient Treatment

To conduct the objective non-inferiority assessment, we needed to estimate the effectiveness of conventional IT in child and adolescent psychiatric care. Given the lack of evidence in the literature (i.e., no existing meta-analysis examined the efficacy of IT vs. control), we performed a systematic search in a first step to obtain the effect size (95% CI) of IT for each primary outcome.

**Question**

To what extent do children, adolescents, and young adults aged 0-21 years with psychiatric disorders benefit from inpatient psychiatric care compared with a control group with respect to clinical outcomes and psychosocial functioning?

**Search string**

(child* OR adolescen* OR teen* OR youth OR young) AND (mental[Title] OR psych*[Title]) AND (inpatient[Title] OR hospital*[Title] OR residential[Title] OR intensive[Title])

**Data bases**

The additional literature search was performed in the same data bases as was the main search of the systematic review: PubMed, CINAHL, PsychINFO, and Embase.

**Criteria for eligibility**

Inclusion: 1) study conducted in humans; 2) English or German language; 3) data reported (original study, meta-analysis, review, …); 4) intervention: psychiatric inpatient treatment or equivalent; 5) CT or RCT (e.g., waitlist control, outpatient control); 6) psychiatric patients with diagnoses according to ICD or DSM; 7) sample age (mean ≤ 21 years)

Exclusion: 1) animal studies; 2) other languages than English or German; 3) no data reported (e.g., letter to the Editor, comment, study protocol …); 4) intervention other than IT (e.g., outpatient treatment, day clinic, online intervention, …); 5) no comparison group/wrong comparison group (e.g., another inpatient treatment group, HT, …); 6) other than psychiatric illness; 7) sample age (mean > 21 years)

**Results of the systematic search on the efficacy of IT**

We found a total of 594 records (529 unique records) of which two met the above inclusion criteria. The PRISMA flowchart in Figure S1 shows a summary of the study selection process. The following studies were included:

1. Green J, Jacobs B, Beecham J, Dunn G, Kroll L, Tobias C, et al. Inpatient treatment in child and adolescent psychiatry-a prospective study of health gain and costs. *J Child Psychol Psychiatry* 2007; **48**: 1259–67.
2. Salzer S, Cropp C, Jaeger U, Masuhr O, Streeck-Fischer A. Psychodynamic therapy for adolescents suffering from co-morbid disorders of conduct and emotions in an in-patient setting: a randomized controlled trial. *Psychol Med* 2014; **44**: 2213–22.


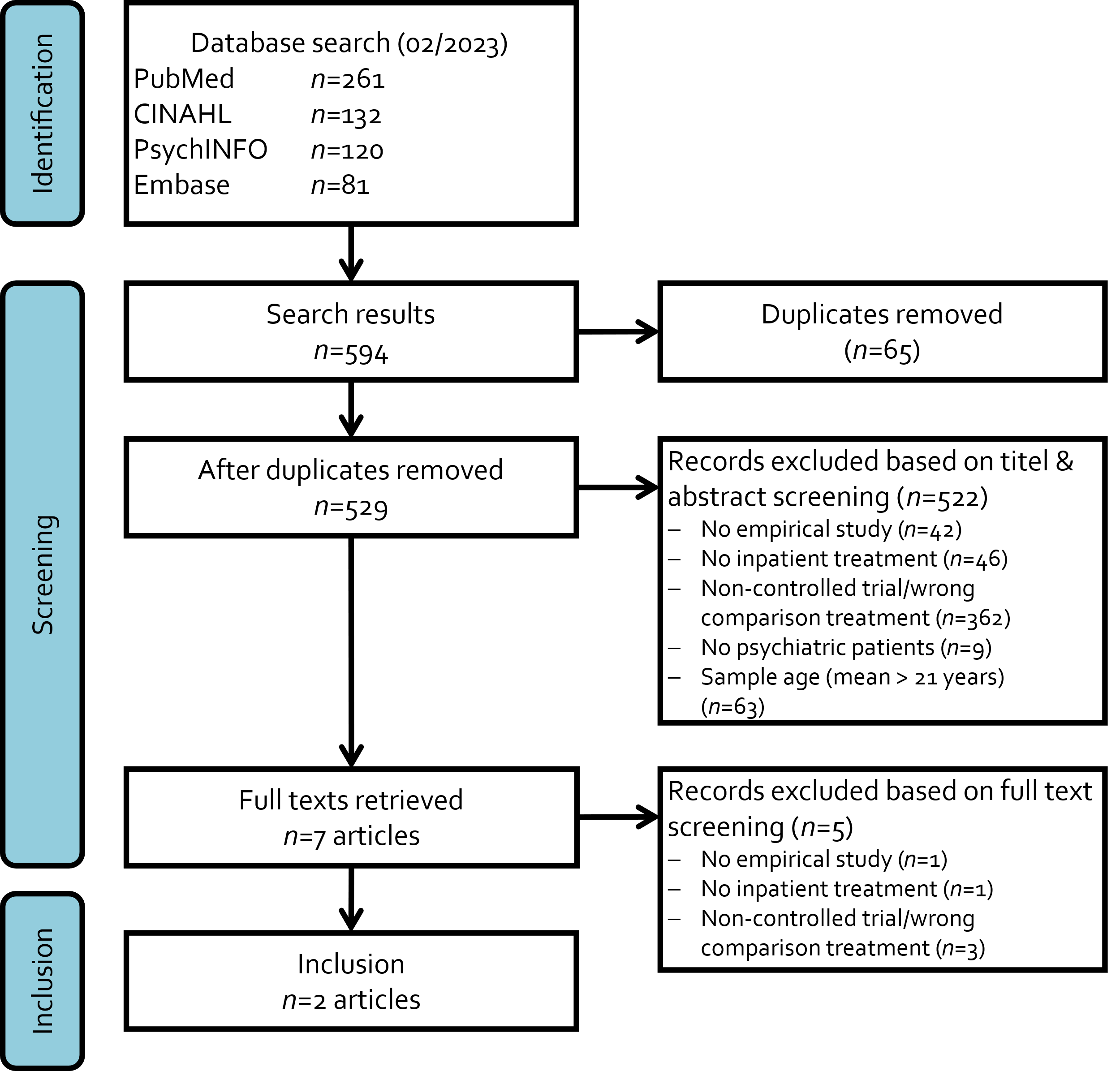


#### **Figure S1**. Prisma flowchart of the additional systematic search

Study selection procedure for publications on the effects associated with conventional child and adolescent psychiatric inpatient treatment compared to an untreated control group.

# Supplementary Results


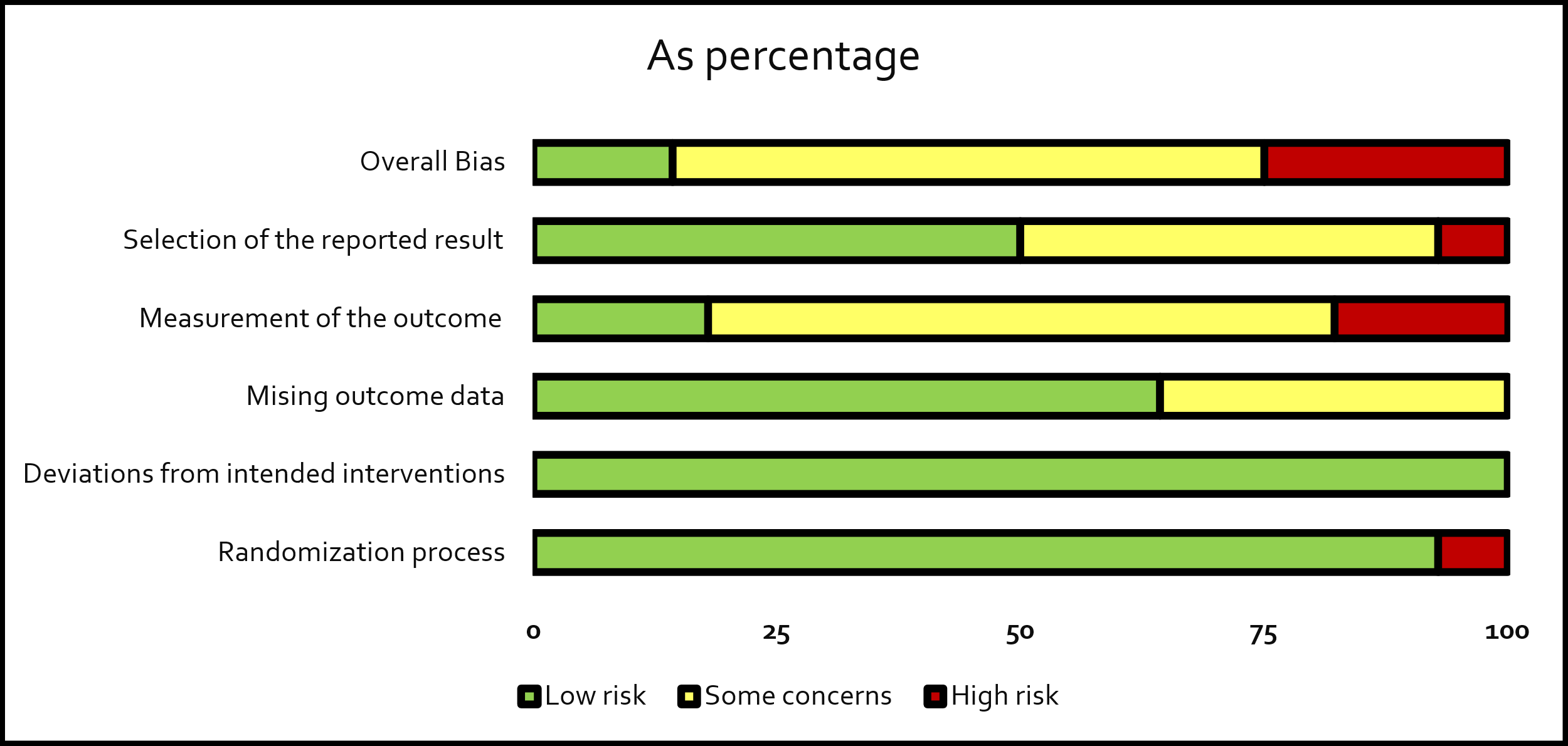


#### **Figure S2.** Summary of the risk of bias of RCTs

Risk of bias assessed using the “Revised Tool to Assess Risk of Bias in Randomized Trials” (RoB 2 [42])


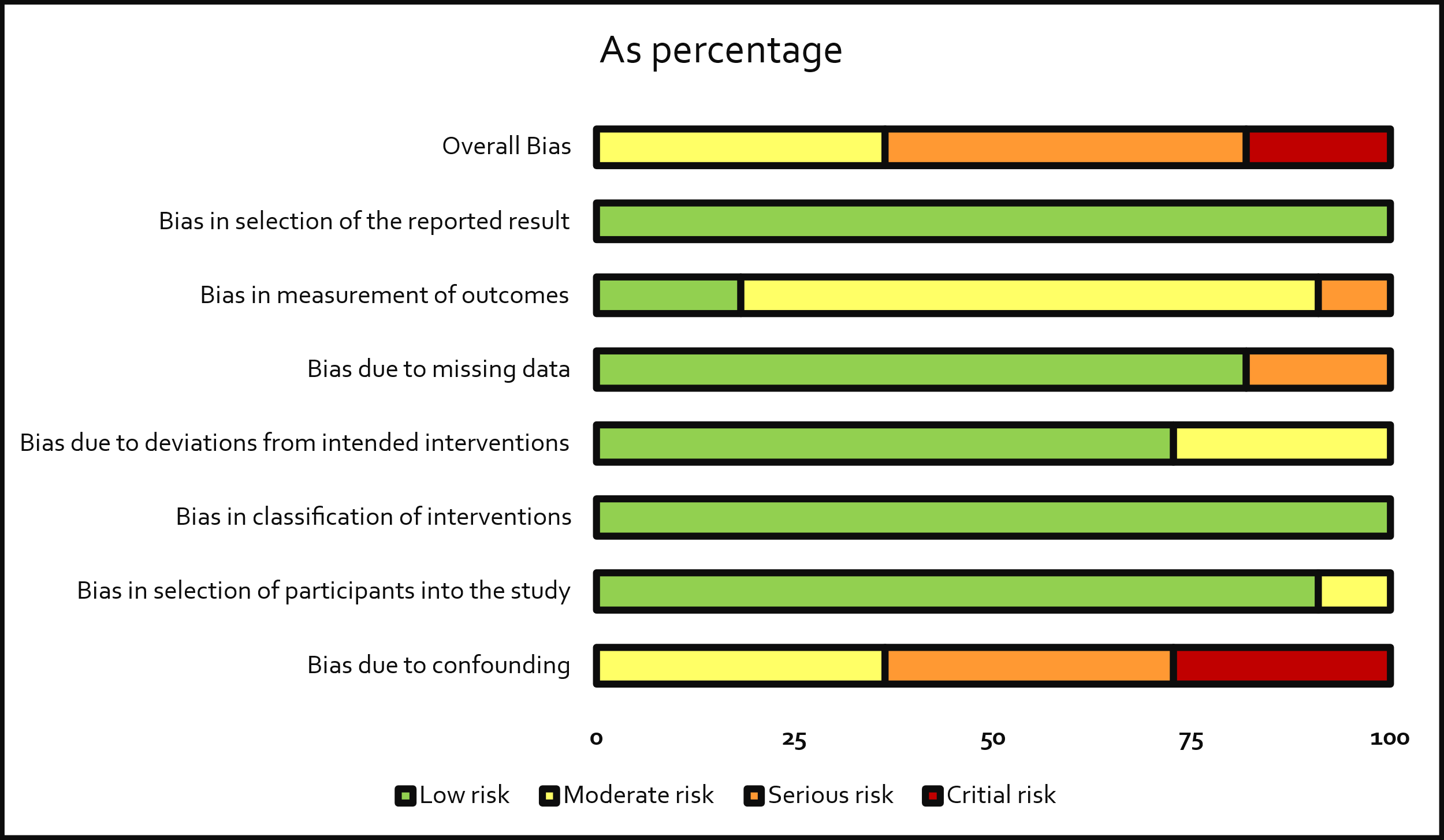


#### **Figure S3**. Summary of the risk of bias of nRCTs

Risk of bias assessed using the “Risk Of Bias In Non-randomized Studies – of Interventions” (ROBINS-I [43]).


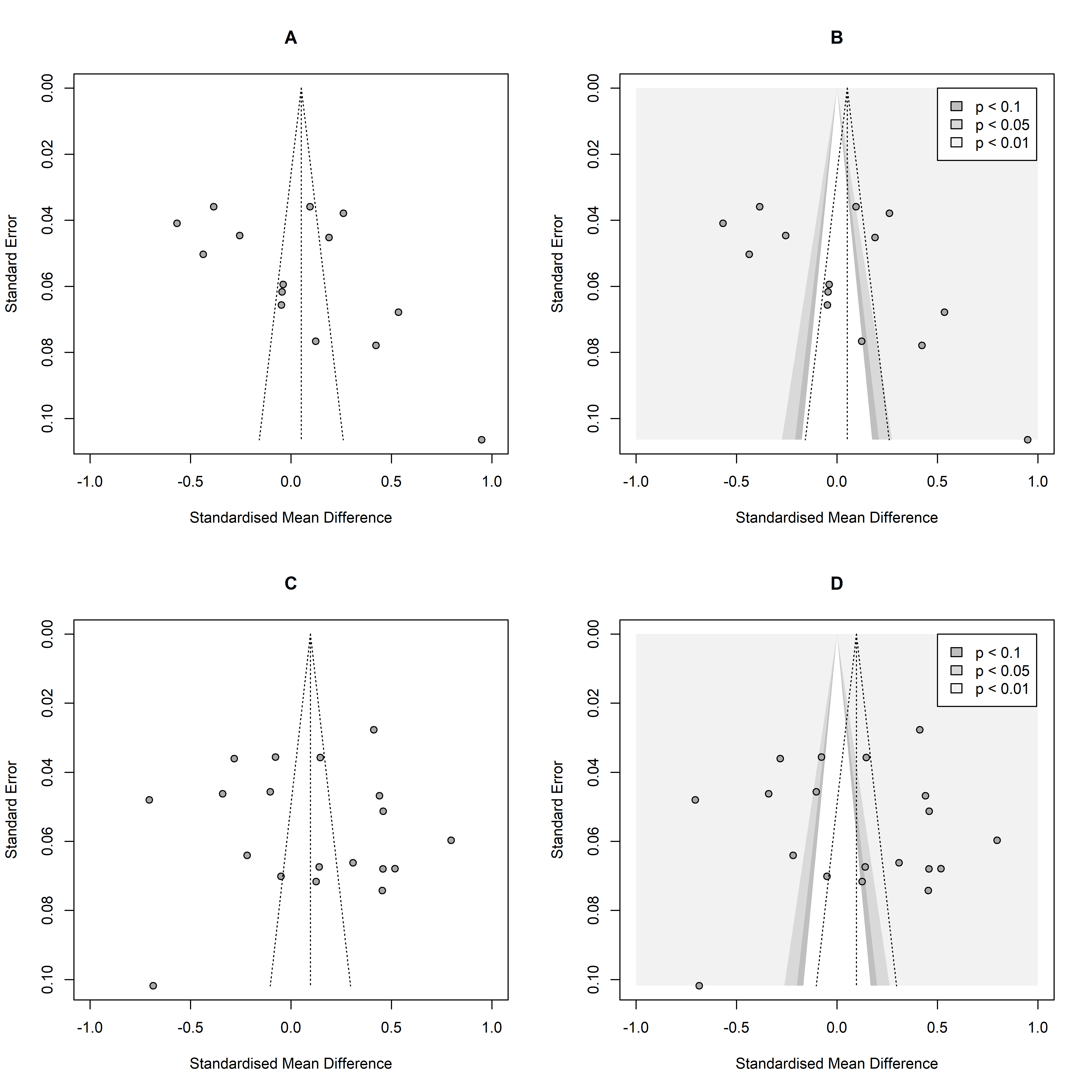


#### Figure S4. Funnel plots of individual observed effect sizes

Plot A & B show effect sizes for post psychosocial functioning, C & D for post psychopathology; A, C: traditional funnel plots centered on the observed summary effect sizes on the x-axis against the corresponding standard errors (i.e., the square root of the sampling variances) on the y-axis. B, D: Contour-enhanced funnel plots centered at zero with grey-shaded regions indicating different levels of statistical significance: The unshaded region in the center of the funnel corresponds to *p*-values greater than .10, the dark grey shaded region corresponds to p-values between 0.10 and 0.05, the light grey shaded region corresponds to *p*-values between 0.05 and 0.01, and the region outside the funnel corresponds to *p*-values below 0.01.

#### **Table S7.** Meta-regression results (conducted for primary outcomes)

| **Moderator** |  |  |  |  |  |  |  |  |  |
| --- | --- | --- | --- | --- | --- | --- | --- | --- | --- |
| **Psychosocial functioning** | **Factor Level(s)** | ***k*** | ***β*** | **SE** | ***p*** | **95% CI:**  **Lower Bound** | **95% CI:**  **Upper Bound** | **QE (*df*)** | ***p*** |
| Age | Continuous (Years) | 13 | -0.06 | 0.06 | .30 | -0.17 | 0.05 | 642.58 | <.001 |
| Sex | Continuous (% Females) | 15 | -0.01 | 0.01 | .16 | -0.02 | 0.004 | 611.26 | <.001 |
| Study design | Binary (RCT; non-RCT) | 15 | -0.31 | 0.24 | 0.20 | -0.78 | 0.16 | 751.05 | <.001 |
| Type of HT | Binary (adjunctive; independent) | 15 | 0.35 | 0.22 | 0.11 | -0.08 | 0.78 | 486.44 | <.001 |
| Mean duration of HT | Continuous (Days) | 14 | 0.001 | 0.003 | .83 | -0.01 | .01 | 602.32 | <.001 |
| Mean duration of IT | Continuous (Days) | 14 | 0.01 | 0.003 | .06 | -0.001 | 0.01 | 472.54 | <.001 |
| Day services | Binary (part of HT; not part of HT) | 15 | -0.35 | 0.21 | 0.10 | -0.76 | 0.06 | 497.44 | <.001 |
| Pooled baseline mean score | Continuous ((M_HT_+M_IT_)/2) | 15 | 0.18 | 0.004 | 0.29 | -0.01 | 0.004 | 744.82 | <.001 |
| Difference in baseline means | Continuous (M_HT_-M_IT_) | 15 | -0.01 | 0.01 | 0.36 | -0.04 | 0.01 | 654.93 | <.001 |
| **Psychopathology** | **Factor Level(s)** | ***K*** | ***β*** | **SE** | ***p*** | **95% CI:  Lower Bound** | **95% CI:  Upper Bound** | **QE (*df*)** | ***p*** |
| Age | Continuous (Years) | 16 | 0.01 | 0.08 | .88 | -0.15 | 0.18 | 798.86 | <.001 |
| Sex | Continuous (% Females) | 19 | 0.01 | 0.01 | .55 | -0.01 | 0.02 | 1061.90 | <.001 |
| **Study design** | **Binary (RCT; non-RCT)** | **19** | **-0.64** | **0.29** | **.03** | **-1.21** | **-0.07** | **869.51** | **<.001** |
| Type of HT | Binary (adjunctive; independent) | 19 | 0.22 | 0.35 | .54 | -0.47 | 0.90 | 946.30 | <.001 |
| Mean duration of HT | Continuous (Days) | 18 | -0.01 | 0.01 | .10 | -0.02 | 0.001 | 874.23 | <.001 |
| Mean duration of IT | Continuous (Days) | 18 | 0.001 | 0.01 | .86 | -0.01 | 0.01 | 760.08 | <.001 |
| Day services | Binary (part of HT; not part of HT) | 15 | -0.23 | 0.31 | 0.44 | -0.83 | 0.36 | 936.23 | <.001 |
| Pooled baseline mean score | Continuous ((M_HT_+M_IT_)/2) | 19 | -0.002 | 0.003 | 0.53 | -0.01 | 0.004 | 1081.27 | <.001 |
| **Difference in baseline means** | **Continuous (M_HT_-M_IT_)** | **19** | **0.10** | **0.03** | **<.001** | **-0.16** | **-0.05** | **502.99** | **<.001** |

Bold font indicates statistical significance of moderators. Analyses were conducted for each moderator separately. If a particular moderator was missing, the corresponding study was excluded from the meta-regression analyses.

Abbreviations: HT=Home Treatment; IT=Inpatient Treatment; SE=Standard error; QE=Test for residual heterogeneity.


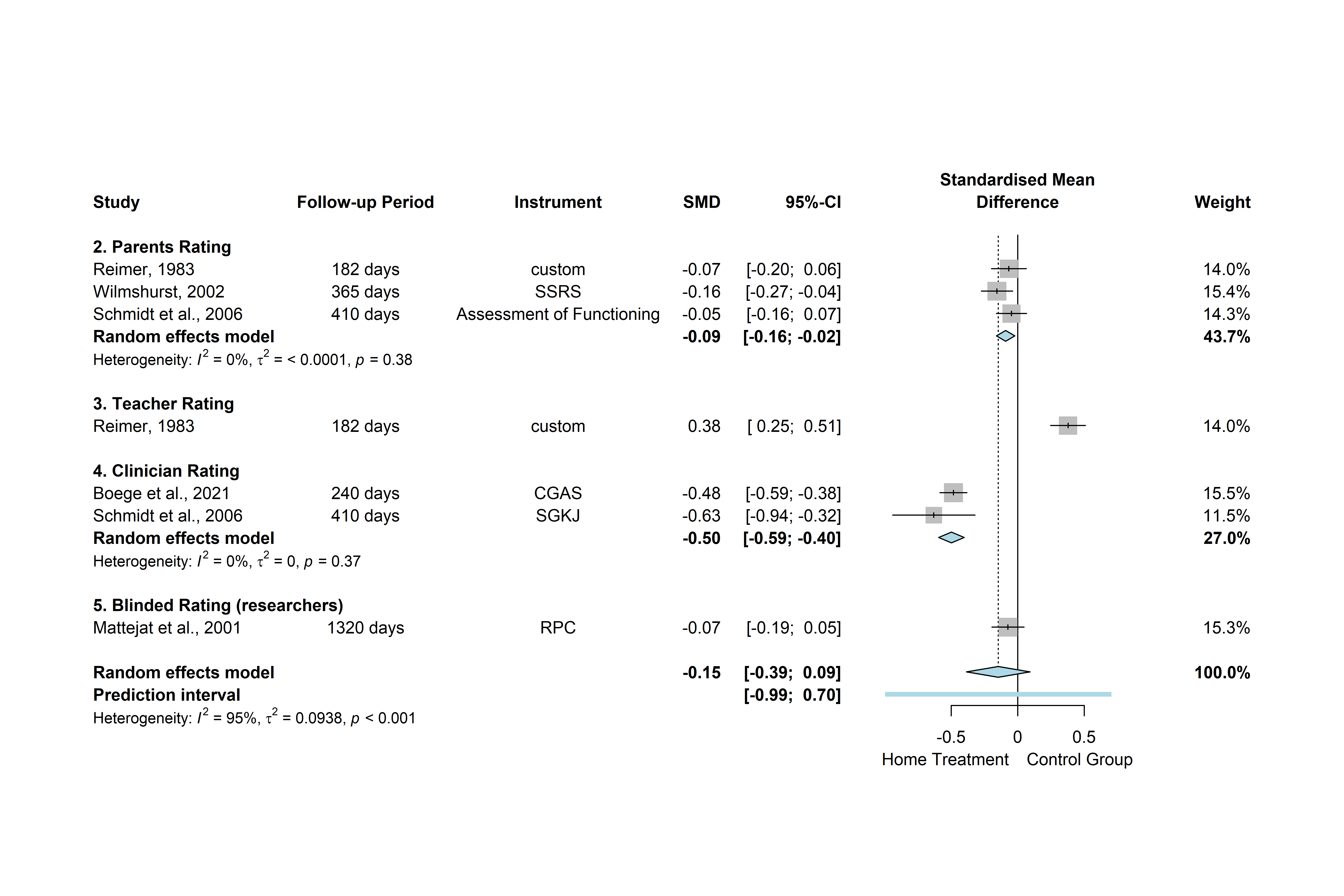


#### **Figure S5**. Difference in pre- to follow-up effects in psychosocial functioning

Abbreviations: SMD=Standardized Mean Difference; CGAS=Children’s Global Assessment Scale; RPC=Rating of psychosocial competency; SGKJ=Global assessment scale for children and adolescents (“Skala zur Gesamtbeurteilung von Kindern und Jugendlichen”).


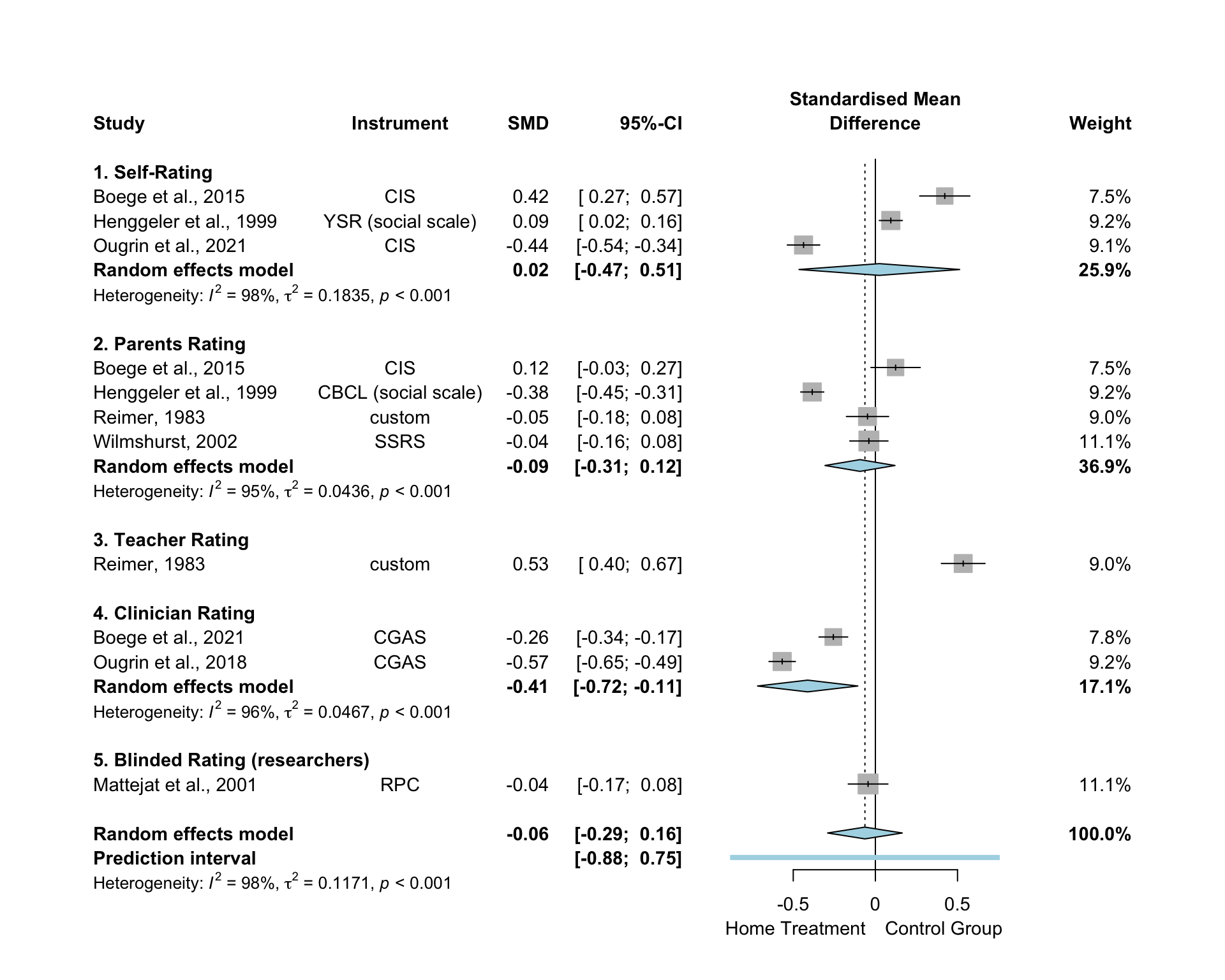


#### Figure S6. Sensitivity analysis: pre- to post-treatment effects in psychosocial functioning, including only Randomised Controlled Trials (RCTs)

SMD=Standardized Mean Difference; CBCL=Child Behaviour Checklist; CGAS=Children’s Global Assessment Scale; CIS=Columbia Impairment Scale; RPC=Rating of psychosocial competency; SSRS=Social Skills Rating System; YSR=Youth Self-Report.


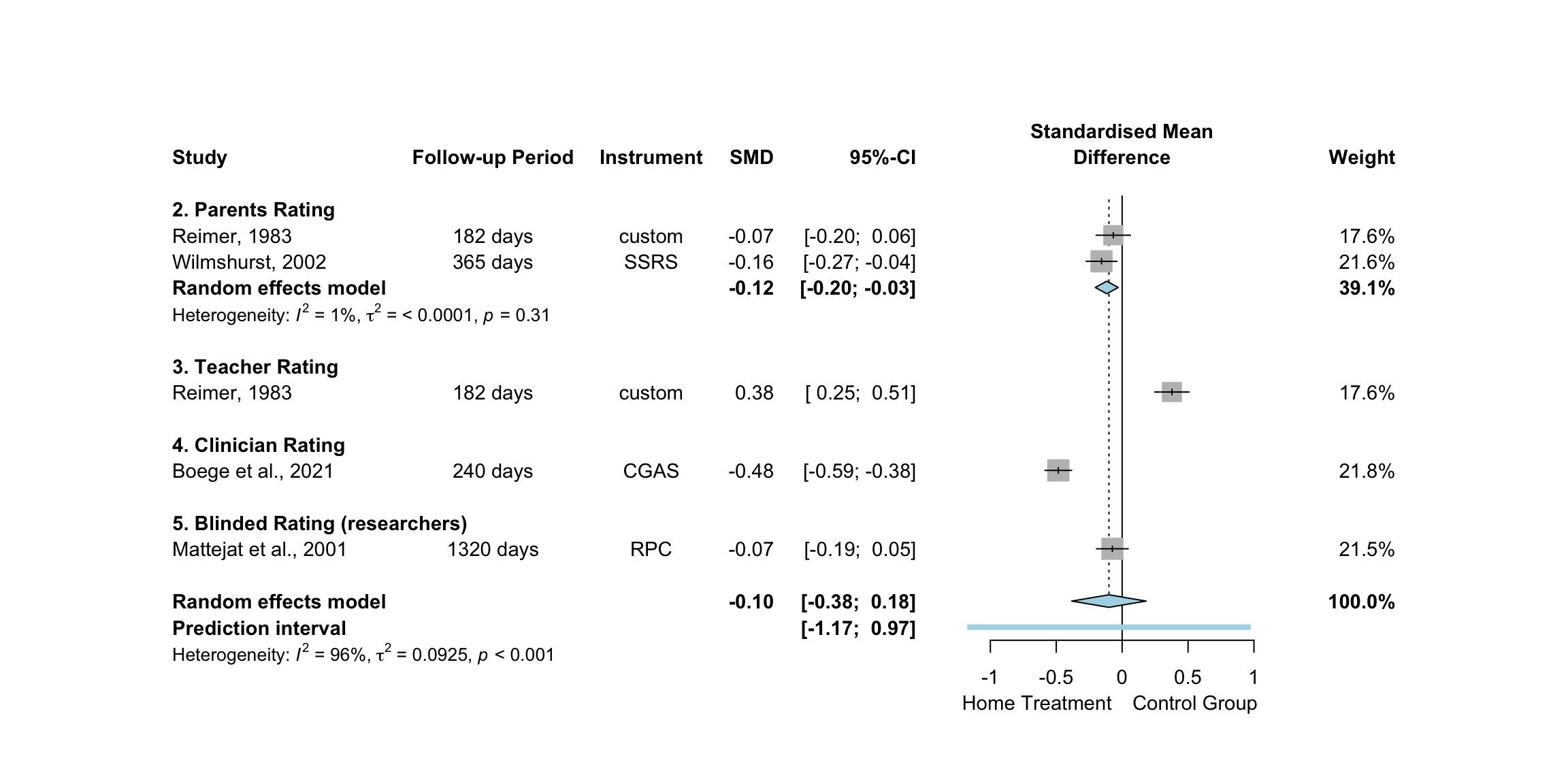


#### Figure S7. Sensitivity analysis: pre- to follow-up effects in psychosocial functioning, including only Randomised Controlled Trials (RCTs)

Abbreviations: SMD=Standardized Mean Difference; CGAS=Children’s Global Assessment Scale; RPC=Rating of psychosocial competency; SSRS=Social Skills Rating System.


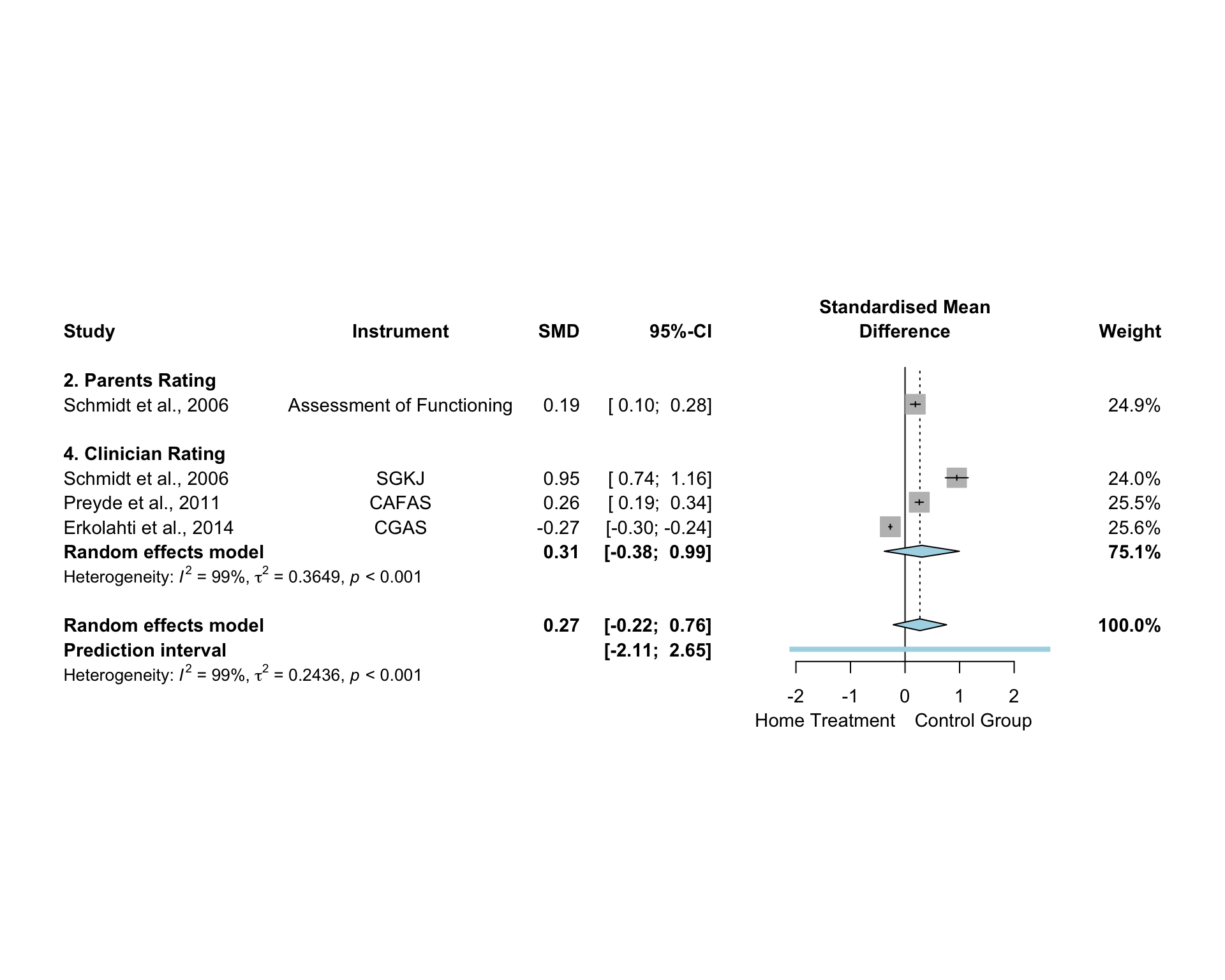


#### Figure S8. Sensitivity analysis: pre- to post-treatment effects in psychosocial functioning, including only non-Randomised Controlled Trials (nRCTs)

Abbreviations: SMD=Standardized Mean Difference; CAFAS= Child and Adolescent Functioning Assessment Scale; CGAS=Children’s Global Assessment Scale; SGKJ=Global assessment scale for children and adolescents (“Skala zur Gesamtbeurteilung von Kindern und Jugendlichen”).


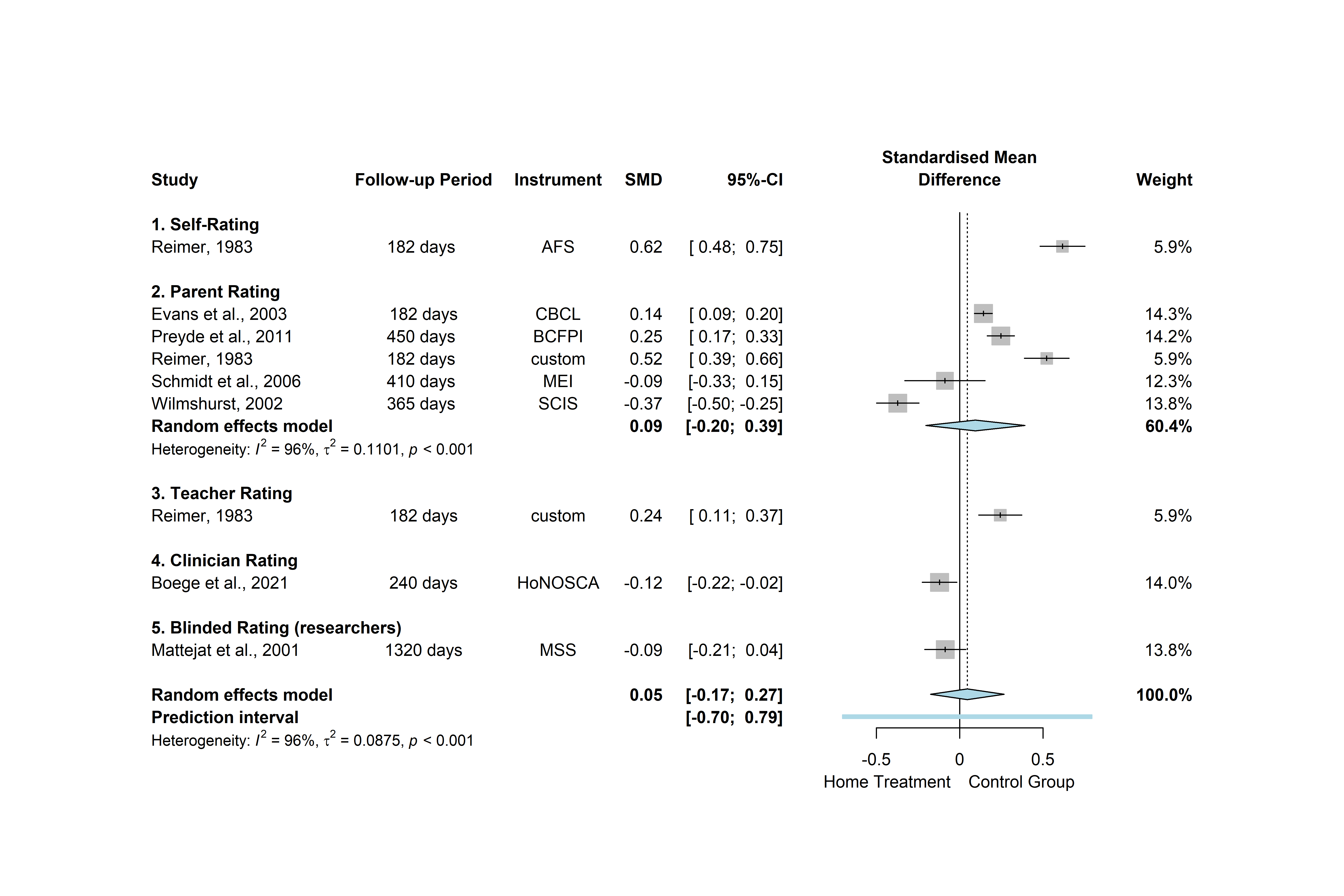


#### **Figure S9.** Difference in pre- to follow-up effects in psychopathology

Abbreviations: SMD=Standardized Mean Difference; AFS=Anxiety questionnaire for pupils (“Angstfragebogen für Schüler”); CBCL=Child Behaviour Checklist; HoNOSCA=Health of the Nations Outcome Scale for children and adolescent; MEI=Mannheim Parents Interview (“Mannheimer Eltern Interview”); MSS=Marburg Symptom Scale; SCIS=Standardized Client Information System.


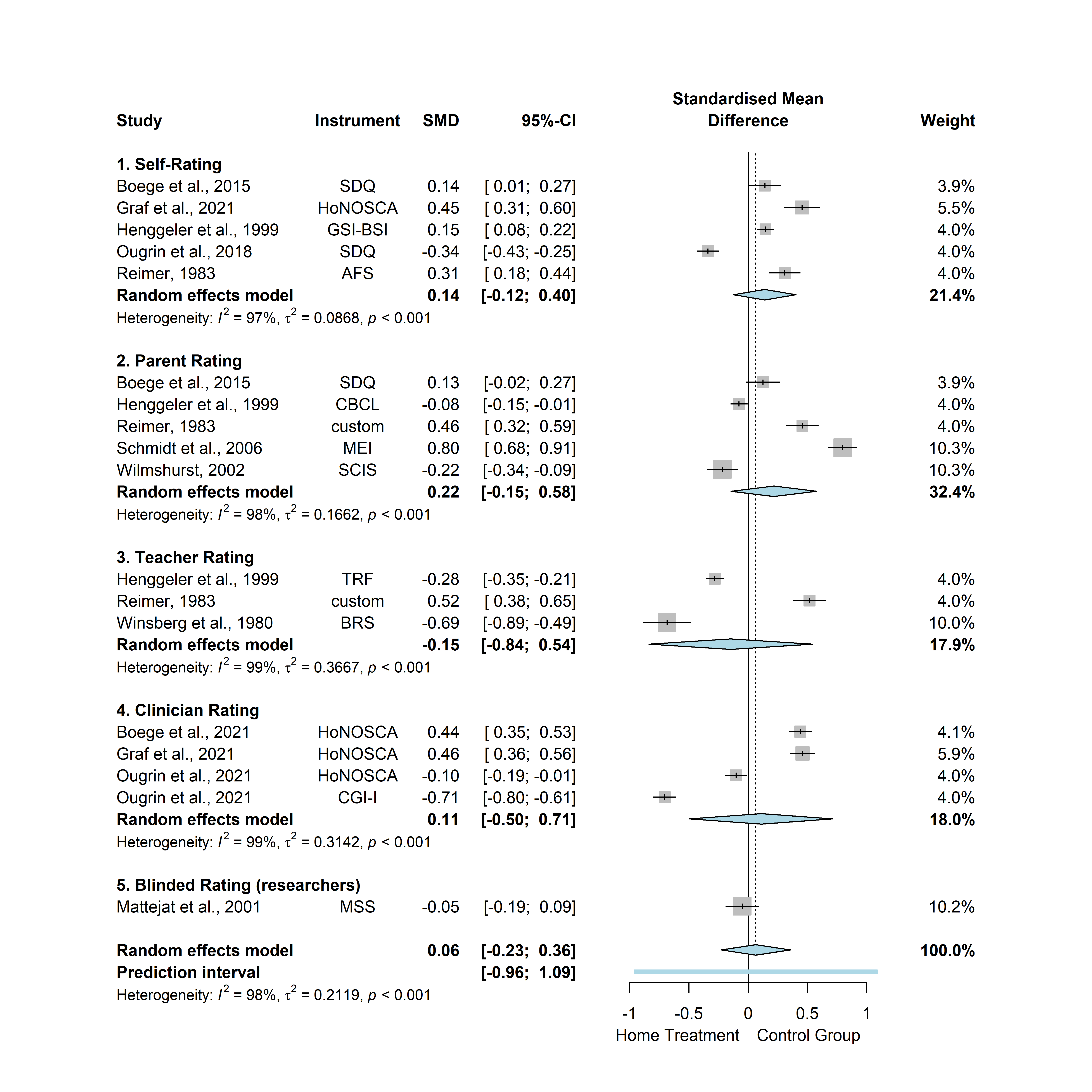


#### **Figure S10.** Sensitivity analysis: pre- to post-treatment effects in psychopathology, excluding the study of Evans et al. (2003) [37]

The study of Evans et al. (2003) [37] compared HT to another alternative for IT (“Crisis Case Management”), which met the formal inclusion criteria but differed substantially from the control condition we intended for comparison. The forest plot shows the difference in pre- to post-treatment psychopathology without the study of Evans et al. (2003).

Abbreviations: SMD=Standardized Mean Difference; AFS=Anxiety questionnaire for pupils (“Angstfragebogen für Schüler”); BRS=Conners Behaviour Rating Scale; CBCL=Child Behaviour Checklist; CGI-I=Clinical Global Impression - Improvement scale; GSI-BSI=Global Severity Index of the Brief Symptom Inventory; HoNOSCA=Health of the Nations Outcome Scale for children and adolescent; MEI=Mannheim Parents Interview (“Mannheimer Eltern Interview”); MSS=Marburg Symptom Scale; SCIS=Standardized Client Information System; SDQ=Strength and Difficulties Questionnaire; TRF=Teacher Report Form.


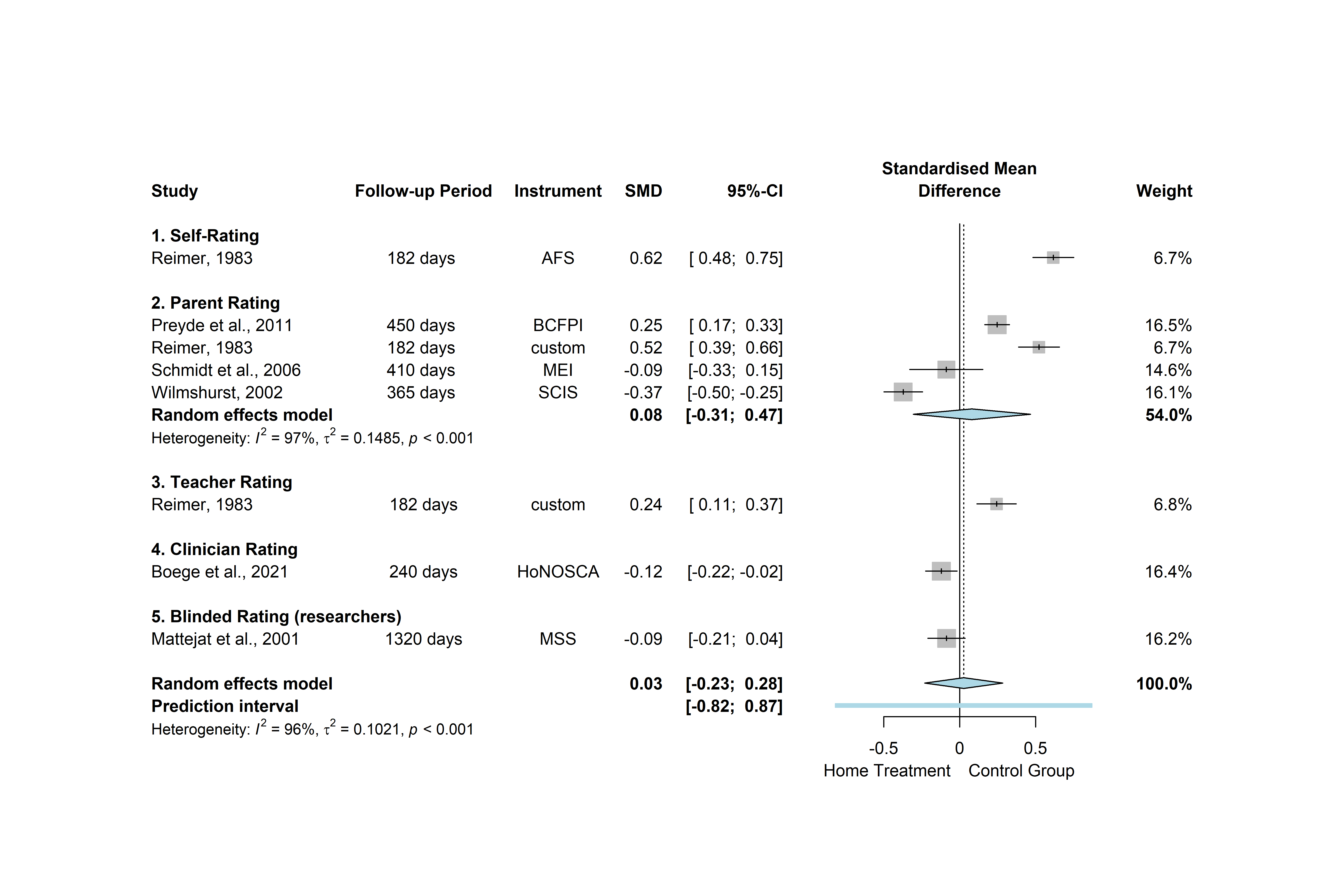


#### **Figure S11.** Sensitivity analysis: pre- to follow-up effects in psychopathology, excluding the study of Evans et al. (2003) [37]

The study of Evans et al. (2003) [37] compared HT to another alternative for IT (“Crisis Case Management”), which met the formal inclusion criteria but differed substantially from the control condition we intended for comparison. The forest plot shows the difference in pre- to follow-up psychopathology without the study of Evans et al. (2003).

Abbreviations: SMD=Standardized Mean Difference; AFS=Anxiety questionnaire for pupils (“Angstfragebogen für Schüler”); BCFPI=Brief Child and Family Phone Interview; HoNOSCA=Health of the Nations Outcome Scale for children and adolescent; MEI=Mannheim Parents Interview (“Mannheimer Eltern Interview”); MSS=Marburg Symptom Scale; SCIS=Standardized Client Information System.


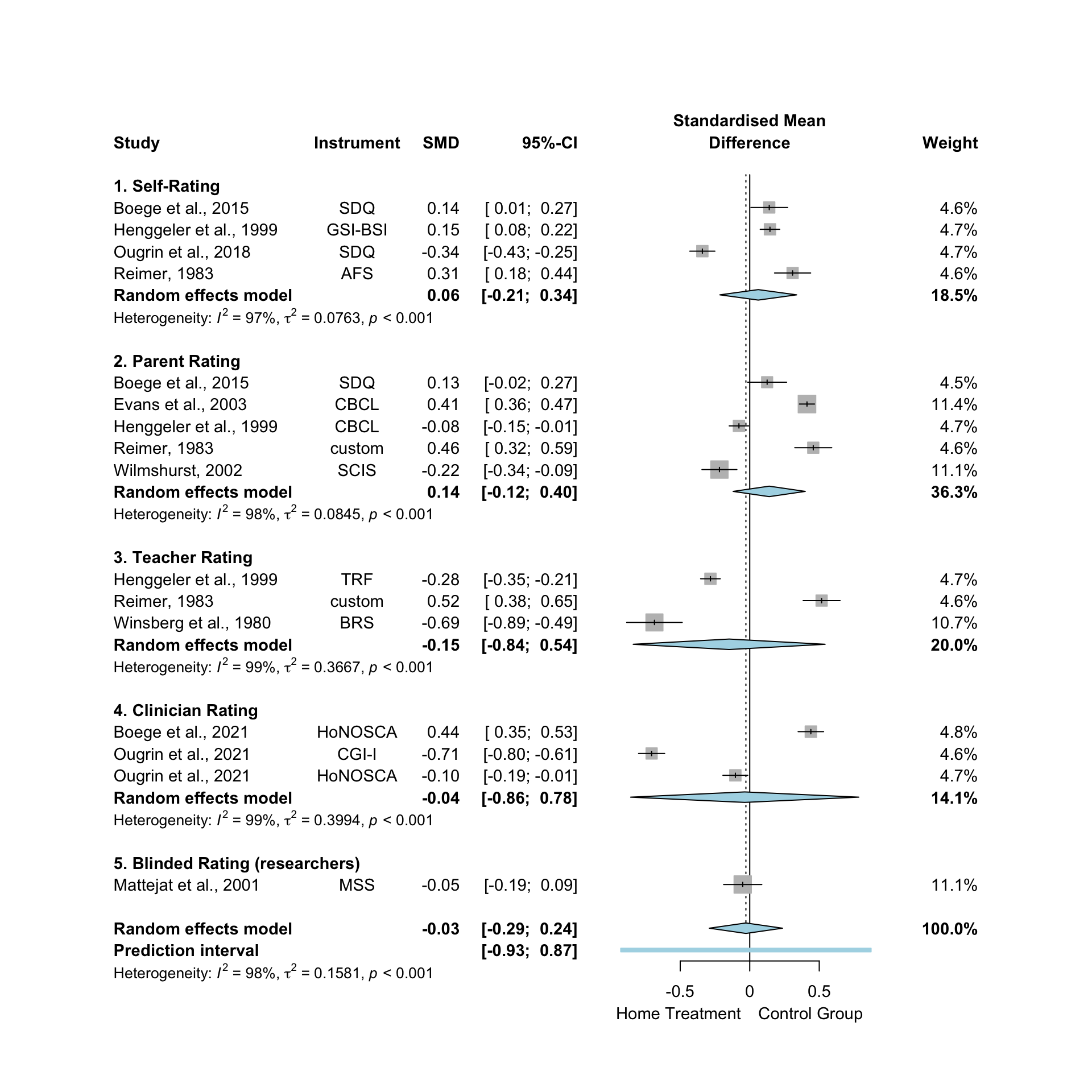


#### Figure S12. Sensitivity analysis: pre- to post-treatment effects in psychopathology, including only Randomised Controlled Trials (RCTs)

Abbreviations: SMD=Standardized Mean Difference; AFS=Anxiety questionnaire for pupils (“Angstfragebogen für Schüler”); BRS=Conners Behaviour Rating Scale; CBCL=Child Behaviour Checklist; CGI-I=Clinical Global Impression - Improvement scale; GSI-BSI=Global Severity Index of the Brief Symptom Inventory; HoNOSCA=Health of the Nations Outcome Scale for children and adolescent; MSS=Marburg Symptom Scale; SCIS=Standardized Client Information System; SDQ=Strength and Difficulties Questionnaire; TRF=Teacher Report Form.


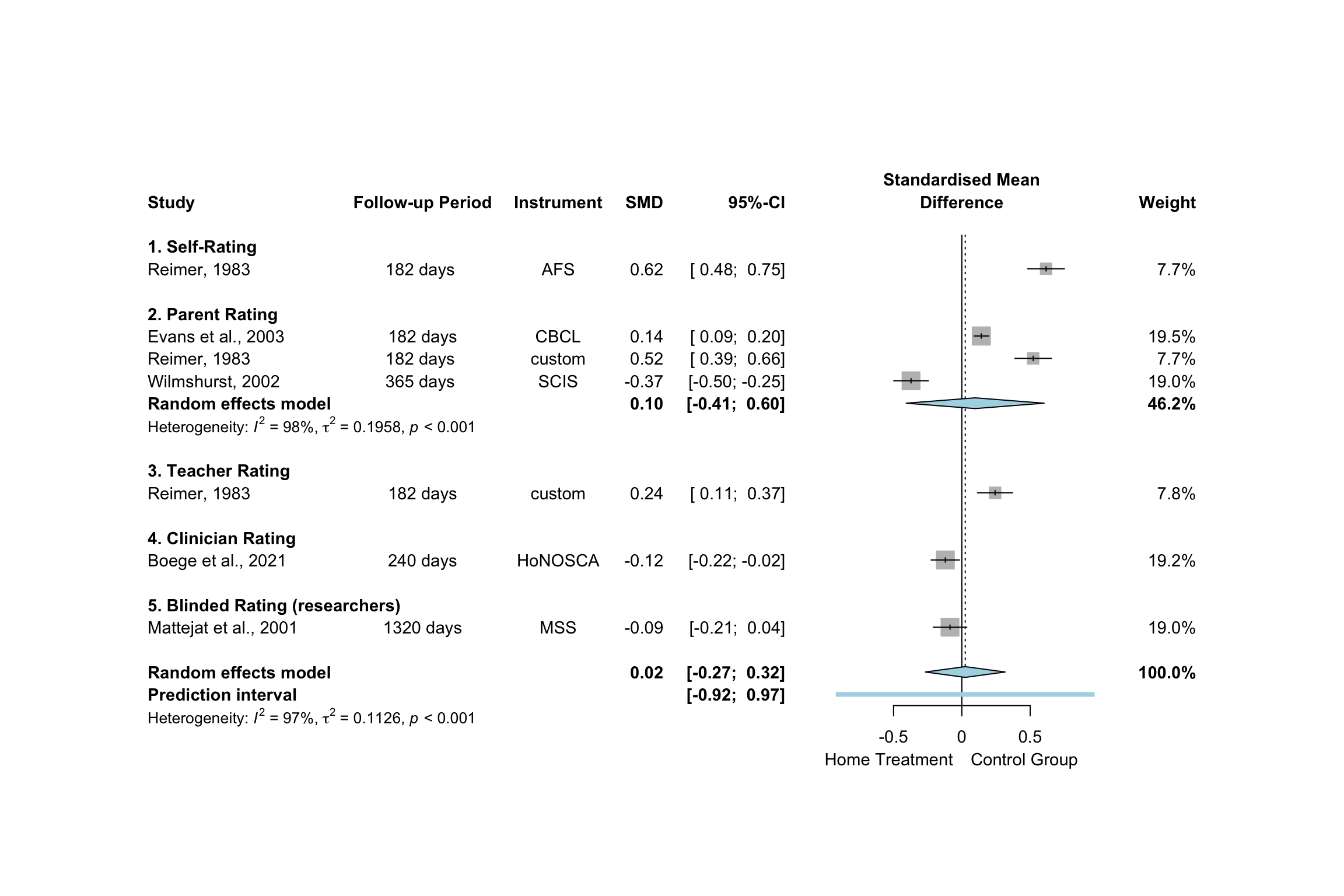


#### Figure S13. Sensitivity analysis: pre- to follow-up effects in psychopathology, including only Randomised Controlled Trials (RCTs)

Abbreviations: SMD=Standardized Mean Difference; AFS=Anxiety questionnaire for pupils (“Angstfragebogen für Schüler”); CBCL=Child Behaviour Checklist; HoNOSCA=Health of the Nations Outcome Scale for children and adolescent; MSS=Marburg Symptom Scale; SCIS=Standardized Client Information System.


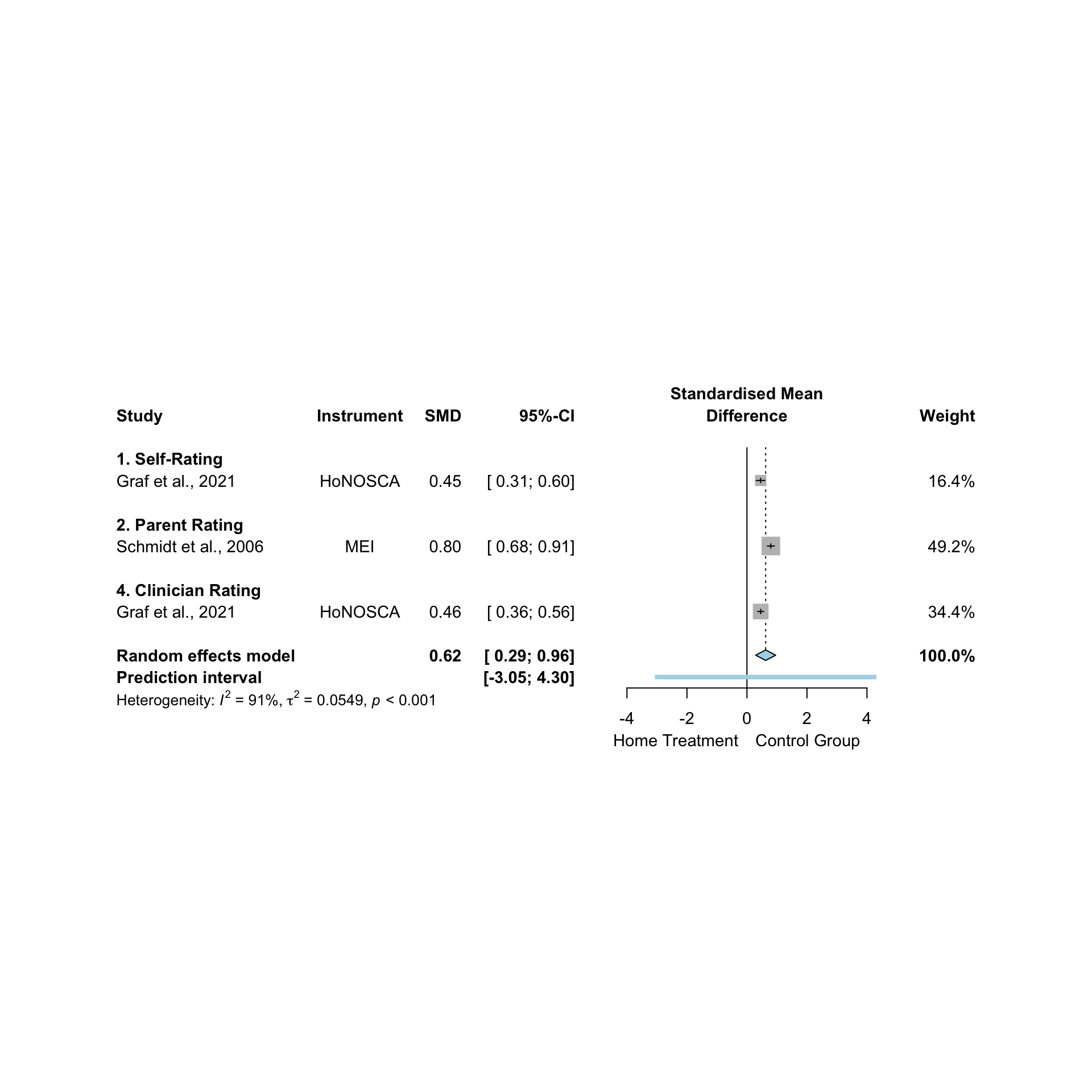


#### Figure S14. Sensitivity analysis: pre- to post-treatment effects in psychopathology, including only non-Randomised Controlled Trials (nRCTs)

Abbreviations: SMD=Standardized Mean Difference; HoNOSCA=Health of the Nations Outcome Scale for children and adolescent; MEI= Mannheim Parents Interview (“Mannheimer Eltern Interview”).


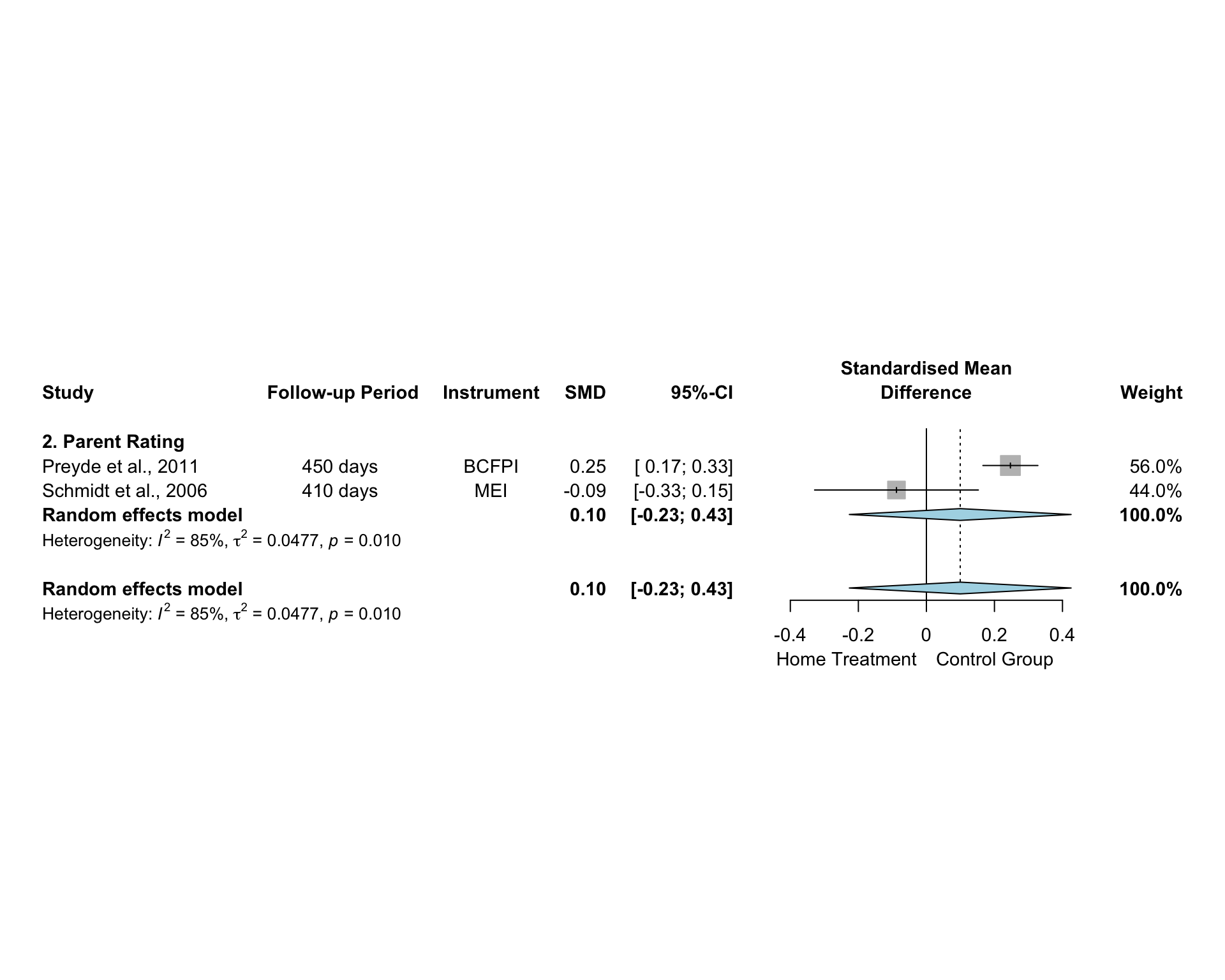


#### Figure S15. Sensitivity analysis: pre- to follow-up effects in psychopathology, including only non-Randomised Controlled Trials (nRCTs)

Abbreviations: SMD=Standardized Mean Difference; BCFPI=Brief Child and Family Phone Interview; MEI= Mannheim Parents Interview (“Mannheimer Eltern Interview”).


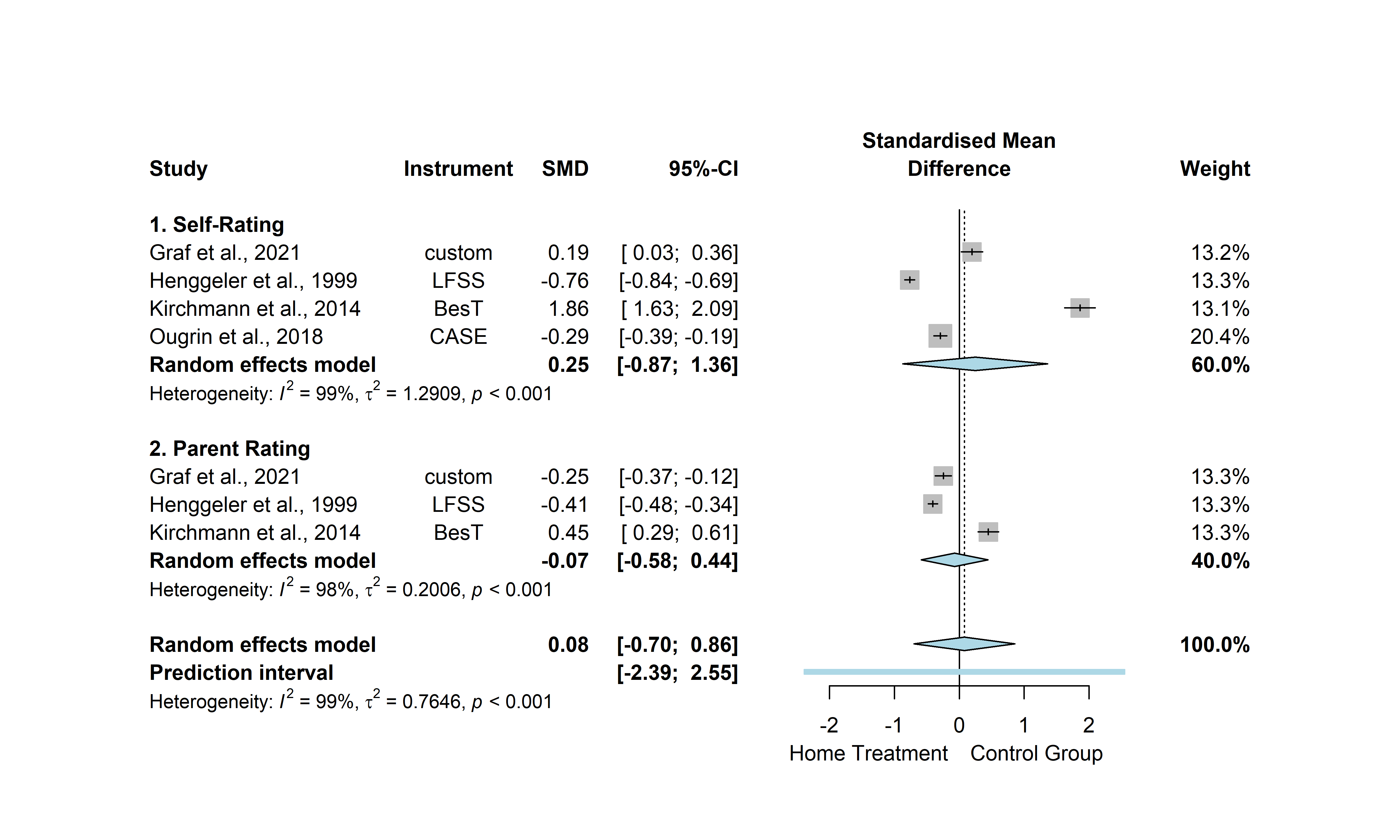


#### **Figure S16.** Difference in treatment satisfaction (post-treatment)

Abbreviations: SMD=Standardized Mean Difference; BesT=Behandlungseinschätzung stationär-psychiatrischer Therapie; ChASE=Child and Adolescent Service Experience; LFSS=Lubrecht’s Family Satisfaction Survey.


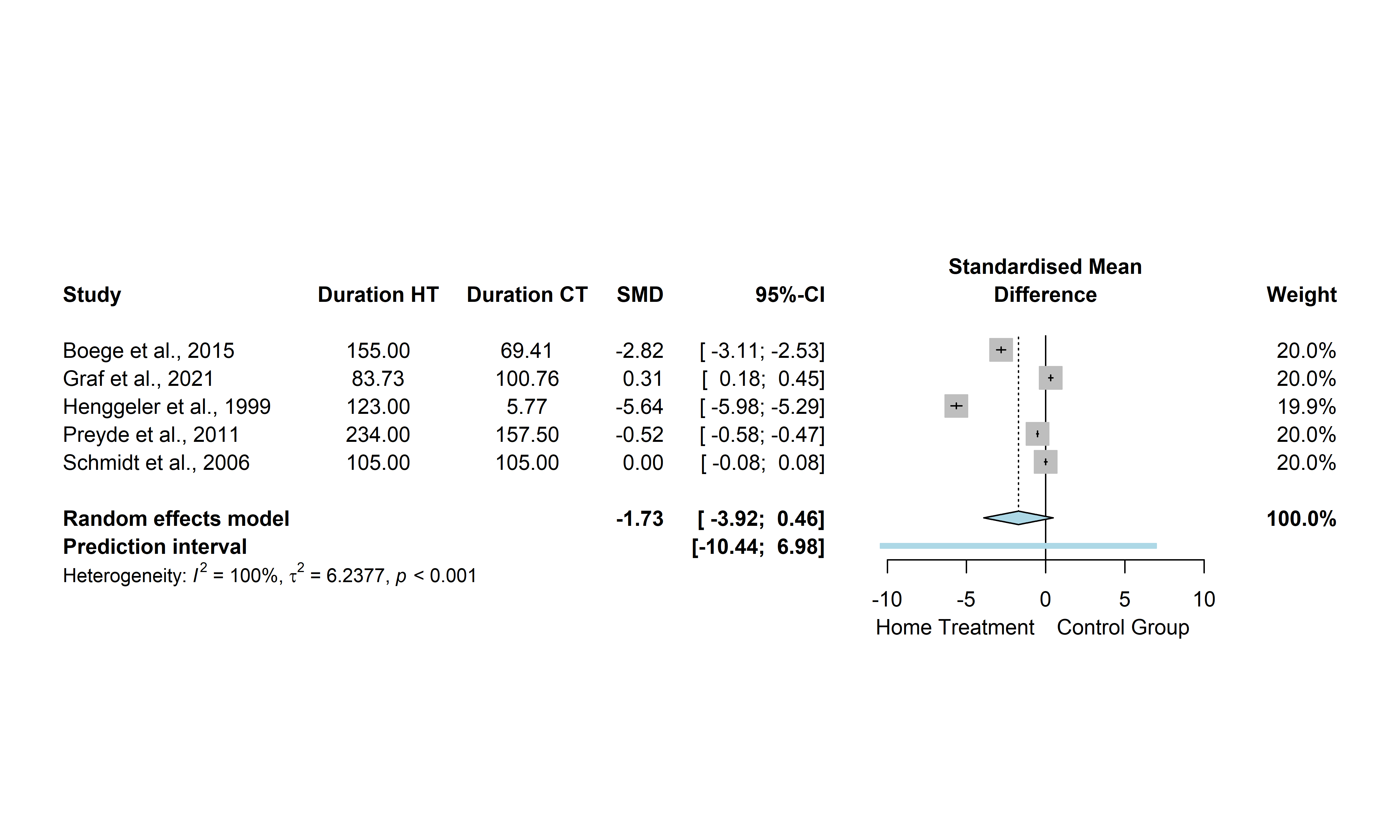


#### **Figure S17.** Difference in treatment duration (in days)

Data from six studies [18, 21, 22, 70, 79, 80] were not considered due to missing standard deviation reporting, which is required to compute SMDs.

Abbreviations: SMD=Standardized Mean Difference.


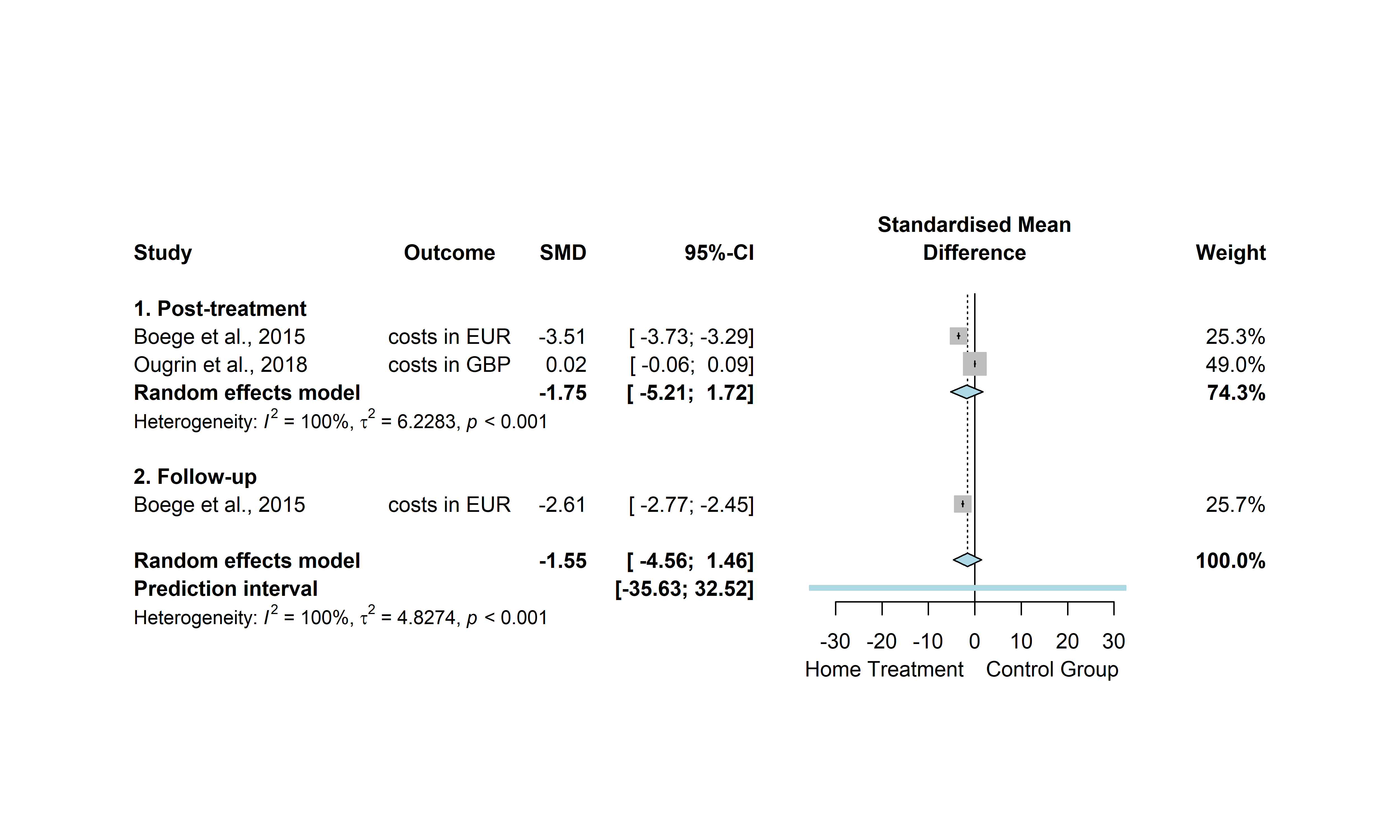


#### **Figure S18**. Difference in treatment costs

1.=direct treatment costs, 2.=costs for the health system generated between discharge and follow-up.

Abbreviations: SMD=Standardized Mean Difference; EUR=€; GBT=£.


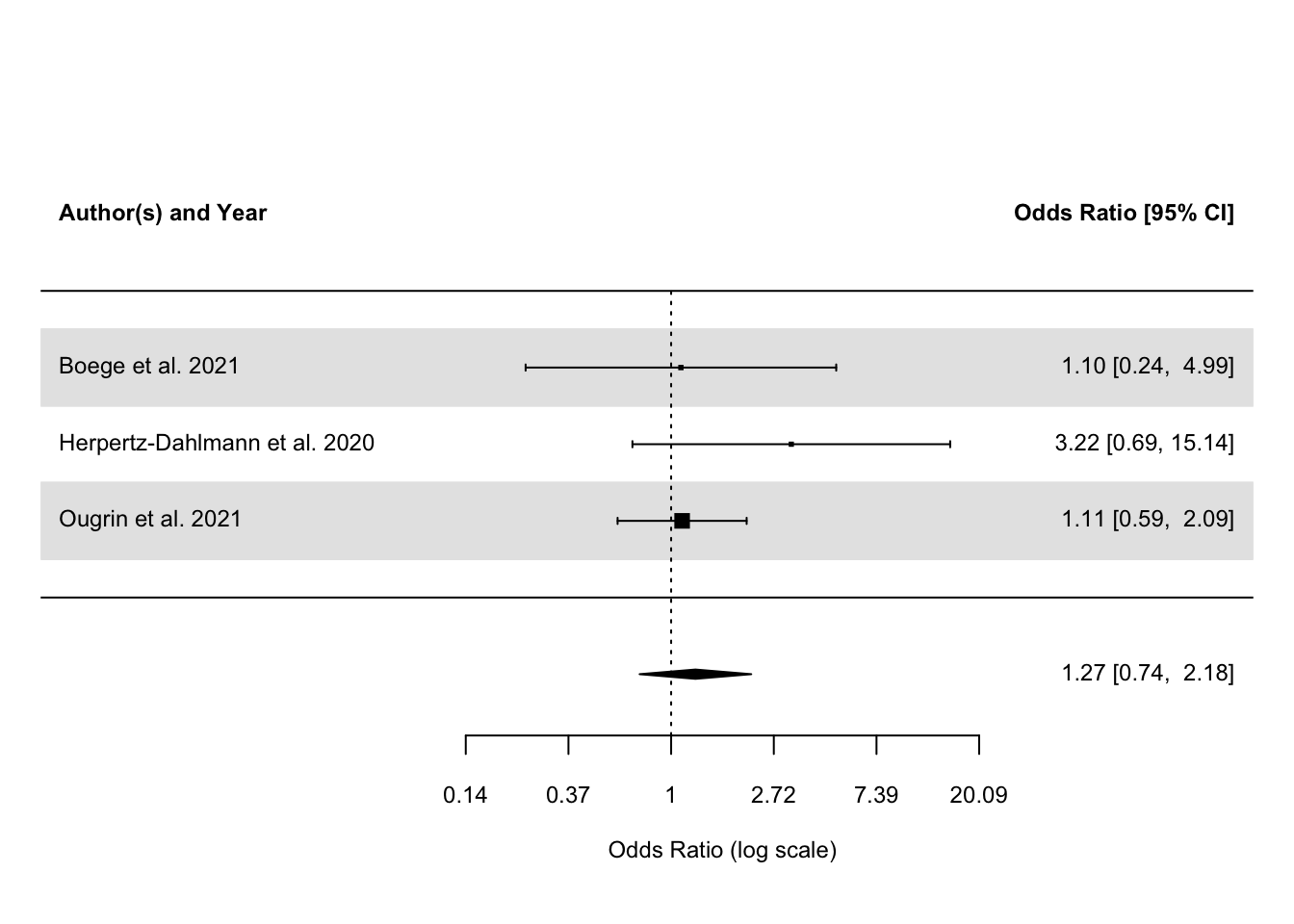


#### Figure S19. Difference in readmission rates post-discharge

Odds ratios above 1 indicate a higher rate of readmission after IT compared to HT, whereas an odds ratio below 1 indicates the opposite


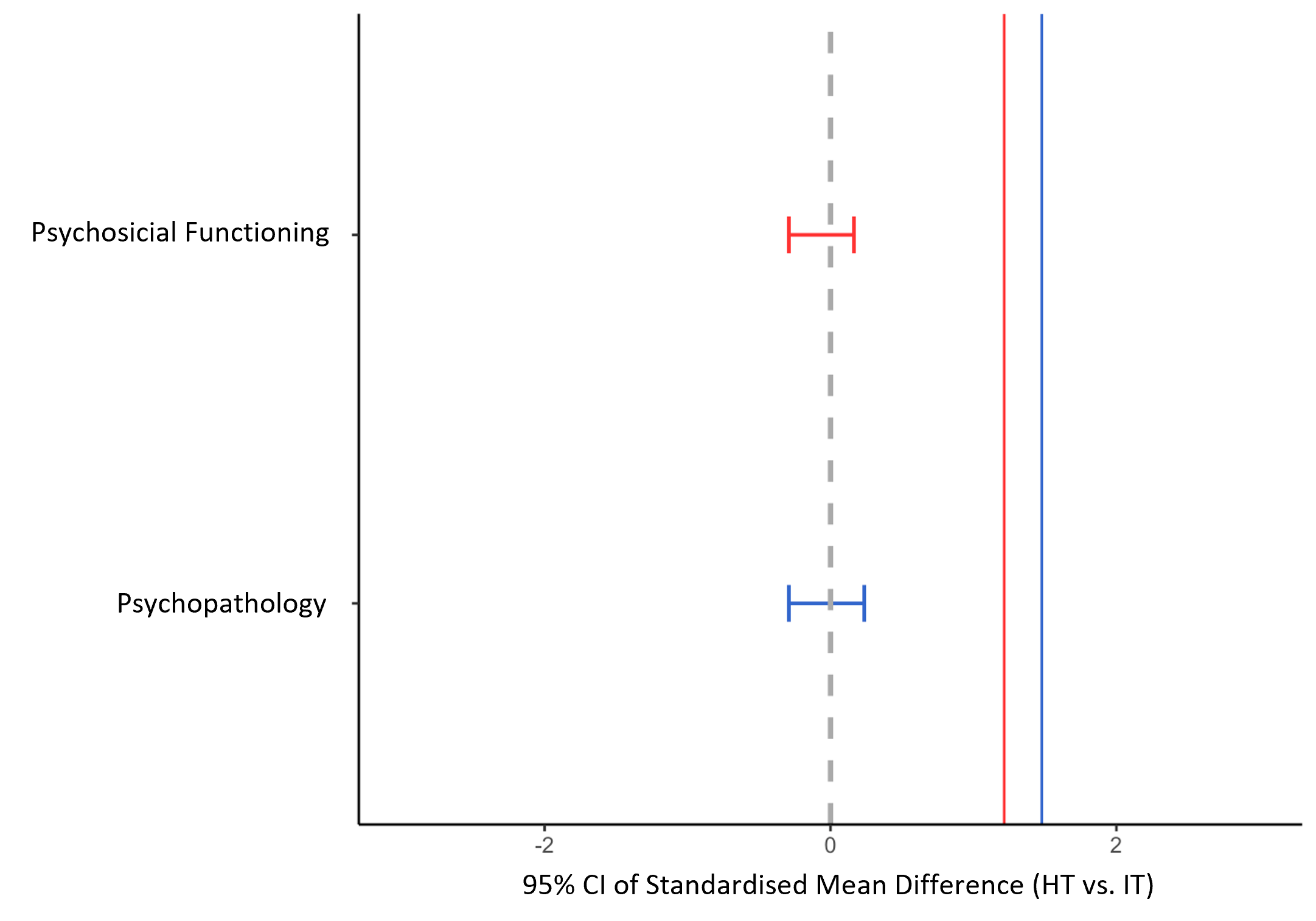


#### Figure S20. Non-inferiority asssessment of Home Treatment (HT) vs. Inpatient Treatment (IT) for Psychosocial Functioning and Psychopathology

The horizontal lines depict the 95% confidence intervals (CIs) of the standardized mean differences (SMDs) between HT and IT. The vertical dashed line indicates the non-inferiority margin (Delta). For non-inferiority to be demonstrated, the entire 95% CI of the HT vs. IT comparison must fall to the left of the Delta line.


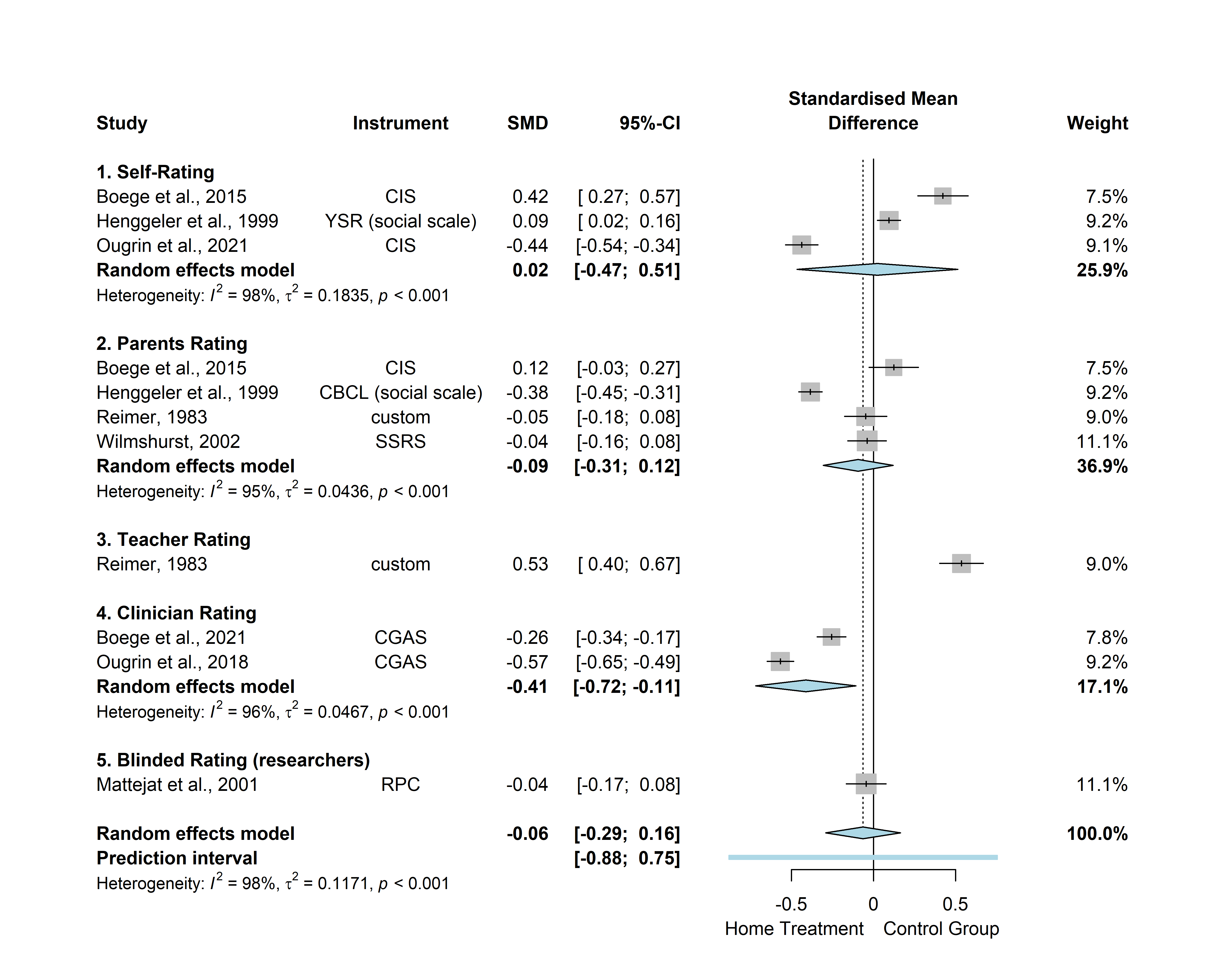


#### Figure S21. Non-inferiority assessment: differences in pre- to post-treatment effects in psychosocial functioning

RCT studies considered only.

Abbreviations: SMD=Standardized Mean Difference; CBCL=Child Behaviour Checklist; CGAS=Children’s Global Assessment Scale; CIS=Columbia Impairment Scale; RPC=Rating of psychosocial competency; SSRS=Social Skills Rating System; YSR=Youth Self-Report.


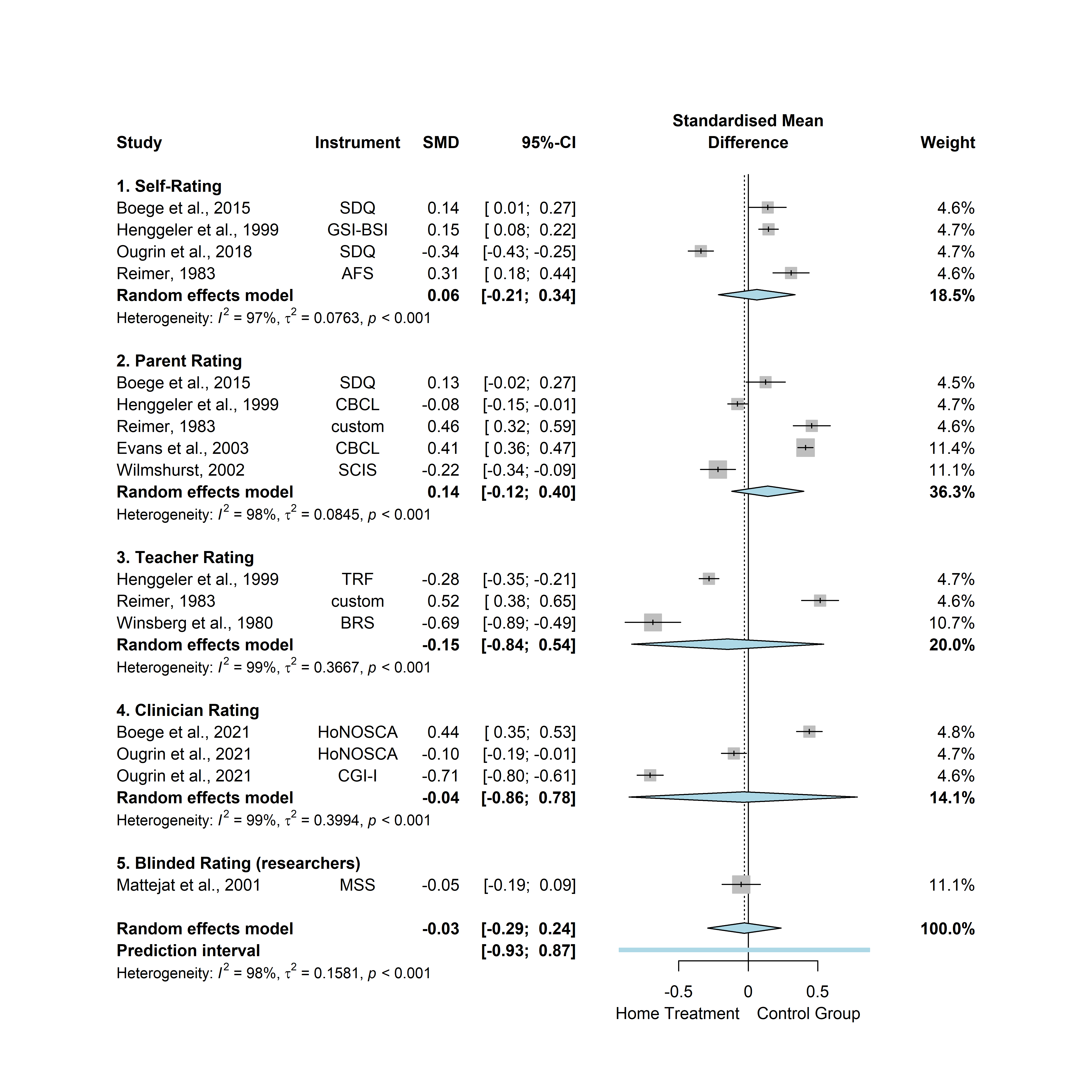


#### **Figure S22**. Non-inferiority assessment: differences in pre- to post-treatment effects in in psychopathology

RCT studies considered only.

Abbreviations: SMD=Standardized Mean Difference; AFS=Anxiety questionnaire for pupils (“Angstfragebogen für Schüler”); BRS=Conners Behaviour Rating Scale; CBCL=Child Behaviour Checklist; CGI-I=Clinical Global Impression - Improvement scale; GSI-BSI=Global Severity Index of the Brief Symptom Inventory; HoNOSCA=Health of the Nations Outcome Scale for children and adolescent; SCIS=Standardized Client Information System; SDQ=Strength and Difficulties Questionnaire; TRF=Teacher Report Form.
